# A Single-Rate Context-Dependent Learning Process Underlies Rapid Adaptation to Familiar Object Dynamics

**DOI:** 10.1371/journal.pcbi.1002196

**Published:** 2011-09-29

**Authors:** James N. Ingram, Ian S. Howard, J. Randall Flanagan, Daniel M. Wolpert

**Affiliations:** 1 Department of Engineering, University of Cambridge, Cambridge, United Kingdom; 2 Department of Psychology and Centre for Neuroscience Studies, Queen's University, Kingston, Ontario, Canada; University College London, United Kingdom

## Abstract

Motor learning has been extensively studied using dynamic (force-field) perturbations. These induce movement errors that result in adaptive changes to the motor commands. Several state-space models have been developed to explain how trial-by-trial errors drive the progressive adaptation observed in such studies. These models have been applied to adaptation involving novel dynamics, which typically occurs over tens to hundreds of trials, and which appears to be mediated by a dual-rate adaptation process. In contrast, when manipulating objects with familiar dynamics, subjects adapt rapidly within a few trials. Here, we apply state-space models to familiar dynamics, asking whether adaptation is mediated by a single-rate or dual-rate process. Previously, we reported a task in which subjects rotate an object with known dynamics. By presenting the object at different visual orientations, adaptation was shown to be context-specific, with limited generalization to novel orientations. Here we show that a multiple-context state-space model, with a generalization function tuned to visual object orientation, can reproduce the time-course of adaptation and de-adaptation as well as the observed context-dependent behavior. In contrast to the dual-rate process associated with novel dynamics, we show that a single-rate process mediates adaptation to familiar object dynamics. The model predicts that during exposure to the object across multiple orientations, there will be a degree of independence for adaptation and de-adaptation within each context, and that the states associated with all contexts will slowly de-adapt during exposure in one particular context. We confirm these predictions in two new experiments. Results of the current study thus highlight similarities and differences in the processes engaged during exposure to novel versus familiar dynamics. In both cases, adaptation is mediated by multiple context-specific representations. In the case of familiar object dynamics, however, the representations can be engaged based on visual context, and are updated by a single-rate process.

## Author Summary

Skillful object manipulation is an essential feature of human behavior. How humans process and represent information associated with objects is thus a fundamental question in neuroscience. Here, we examine the representation of the mechanical properties of objects which define the mapping between the forces applied to an object and the motion that results. Knowledge of this mapping, which can change depending on the orientation with which an object is grasped, is essential for skillful manipulation. Subjects performed a virtual object manipulation task by grasping the handle of a novel robotic interface which simulated the dynamics of a familiar object which could be presented at different orientations. Using this task, we show that adaptation to the properties of a particular object is extremely rapid, and that such adaptation is confined to the specific orientation at which the object is experienced. Moreover, the pattern of adaptation observed when the orientation of the object and its mechanical properties were changed from trial-to-trial was reproduced by a model which included multiple representations and a generalization function tuned for object orientation. These results suggest that the skillful manipulation of objects with familiar dynamics is mediated by multiple context-specific representations.

## Introduction

Object manipulation is an essential feature of everyday human behavior [1]. It represents a challenge for the motor system because grasping an object changes the relationship between the motor commands and the resulting movement of the arm [2], [3], [4]. Skillful manipulation thus requires the rapid adaptation of motor commands to the particular dynamics of the object. This adaptation can be facilitated by using stored knowledge such as an internal model of object dynamics [5], [6], [7], [8]. Previous studies have examined the representation of dynamics using tasks in which subjects adapt their reaching movements to novel and unusual force-fields applied to the hand by robotic interfaces [9], [10], [11], [12], [13], [14]. In these tasks, the force-field alters the normal dynamics of the arm, inducing movement errors which reduce gradually across many trials. Several models have been developed to explain how errors on each trial result in the gradual acquisition of an internal representation of the perturbing dynamics [15], [16], [17], [18], [19], [20]. This approach is based on state-space models in which, typically, the internal state represents an estimate of the perturbation. The state estimate is updated after each trial based on the error experienced on the previous trial. Recently, a model which includes two internal states has been proposed [17]. The two states adapt independently at different rates (one fast, one slow) and sum to produce an estimate of the perturbation. Importantly, this dual-rate model can reproduce phenomena observed experimentally that a single-rate model cannot [17], [19], [ for an alternative view see 21]. State-space models have also been used to account for generalization, in which the movement direction (the kinematic context) varies across trials [15], [19]. In this case, each movement direction has its own state in the model, representing the estimate of the perturbation associated with a movement in that direction. A generalization function specifies how an error experienced during a movement in one direction affects the states associated with other directions. These models can successfully reproduce the trial-by-trial changes in performance and the pattern of generalization observed during tasks that involve novel dynamics and visuomotor perturbations.

The novel dynamics described above typically take subjects tens to hundreds of trials to learn [22]. In contrast, when interacting with everyday objects subjects adapt to the familiar dynamics much more rapidly. For example, when lifting an object of unknown mass, subjects adapt their predictive load and grip forces within just a few trials [5], [23], [24], [25], [26], [27], [28], [29]. Given appropriate visual and other contextual cues subjects can even generate appropriate motor commands on the very first trial [5]. Rapid adaptation of grip force is also observed during bimanual object manipulation, when subjects pull on an object with one hand while stabilizing it with the other [30], [31]. In this case, grip force adaptation can be shown to be context-specific, being locally confined to the movement direction in which the object is experienced [31]. As such, adaptation to the dynamics associated with familiar objects also appears to manifest context-dependent generalization. Moreover, the adaptation, while rapid, nevertheless occurs progressively as a result of trial-by-trial errors associated with an internal estimate of object parameters. This suggests that context-dependent state-space models may also be applicable to adaptation associated with manipulating objects with familiar dynamics.

**Figure 1:**
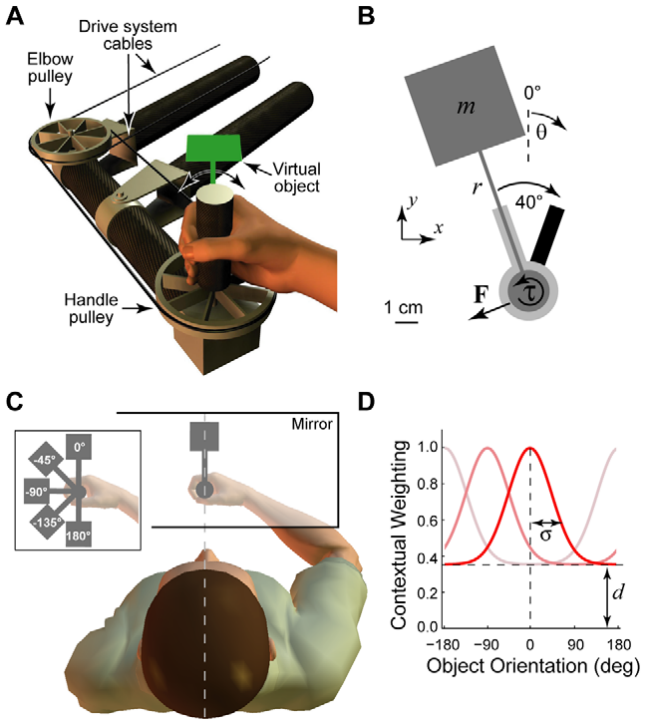
The robotic manipulandum and virtual object manipulation task. **A** The WristBOT is a planar two-dimensional robotic manipulandum which includes torque control at the vertical handle. Cables and pulleys (two are shown) implement the transmission system between the handle and the drive system at rear of manipulandum (not shown). Subjects grasped the vertical handle of the WristBOT and manipulated a virtual object (shown in green). **B** The object dynamics were simulated as a point mass (*m*) on the end of a rigid rod (length *r*) of zero mass. Visual feedback of the object was updated in real-time (dark grey). The task involved rotating the object 40° from a starting angle (light grey bar) to a target angle (black bar) while maintaining the handle within a circular home region (light grey disc). Rotation generated translational forces (**F**) and rotational torques (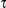) at the handle. Figure shows a grey-scale version of actual visual feedback (see scale bar). Annotations have been added. **C** Top view showing visual feedback of the virtual object (dark grey), which was projected over the subject's hand in the plane of movement. Visual feedback was consistent with grasping the object at its base. The WristBOT handle translates in the horizontal plane and rotates around the vertical axis. Subjects viewed visual feedback in a mirror which prevented them from seeing either their hand or the manipulandum. Dotted line shows subject's mid-sagittal plane which was aligned with the hand and the vertical rotation axis of the object. Inset shows top view of subject's hand overlaid with five different visual orientations of the object. **D** The Gaussian tuning function (see Equation 11, 12 and 13) which implemented the context-selection vector in the MCSRM4. The function has two parameters which describe the standard deviation of the Gaussian (*σ*) and its offset (*d*). The function is centered on the current orientation of the object (0° for the red line) and takes the value of 1 at this point. The function decays to *d* (the offset) at ±180° relative to the current orientation. The pink and pale pink lines show the tuning function for −90° and 180°, respectively.

In a previous study, we examined adaptation to familiar object dynamics by presenting subjects with a virtual hammer-like tool [32]. The task involved rotating the object while keeping the grasp point stationary (Figure 1A to C). This required subjects to generate a torque to rotate the object as well as a force to stabilize the grasp point. The dynamics were simulated using a novel robotic manipulandum (the WristBOT [33]) which can produce forces and torques that depend on the translational position and angular rotation of its vertical handle. The visual orientation of the object could also be varied from trial to trial. Results showed that subjects generate anticipatory forces in the direction appropriate for the visually-presented orientation of the object, even before they had been exposed to the dynamics. This suggests that subjects have pre-existing knowledge of the structural form of the dynamics that can be recalled based on vision. Moreover, when exposed to the dynamics of a specific object, subjects rapidly adapt the magnitude of their anticipatory forces over the first few trials to be appropriate for the particular mass of the object. To probe the representation of the dynamics, we examined the force magnitude at novel visual orientations of the object, where the dynamics had not been experienced. Consistent with previous studies, both of novel dynamic perturbations and familiar object dynamics, we showed that generalization of force magnitude was context-specific, being limited to orientations close to those at which the object had been experienced. However, in contrast to previous studies, the kinematics of the movements in our task were unchanged, and the pattern of generalization observed depended only on the visual context (the orientation) of the object.

The aim of the current study was to determine whether a state-space model could account for rapid context-specific adaptation to the familiar dynamics of everyday objects and whether such adaptation would be mediated by a single-rate or dual-rate process. Specifically, we developed a state-space model that includes multiple context-dependent states and a generalization function tuned to the visual orientation of the object. The results show that a single-rate context-dependent model reproduces the time-course of adaptation and de-adaptation, along with the context-specific behavior observed in three experiments from our previous study. In these previous experiments, subjects were exposed to the dynamics of the object at a single orientation. The model also makes predictions with regards to exposure at multiple orientations which we confirm in two new experiments.

## Methods

### Ethics statement

The study was approved by the local ethics committee and 68 subjects provided informed consent before participating.

A preliminary analysis of experiments 1, 2 and 3 has been previously reported [32]. We include the basic methods for these experiments here for completeness. In the current study, we extend our previous analysis, run additional subjects on a new condition in Experiment 2, and perform three new experiments (4, 5 and S1). In addition, we apply state-space models to the data from all experiments.

### The task

Subjects were seated at a virtual reality system and grasped the handle of a planar robotic manipulandum (the WristBOT) with their right hand (Figure 1A). The WristBOT can generate translational forces in the horizontal plane and a rotational torque about its vertical handle [33]. This allowed us to provide haptic feedback (forces and torques) of the dynamics of a simulated object (Figure 1B). A virtual reality display system provided visual feedback associated with the object and the task (Figure 1C). The object was a small hammer-like tool which consisted of a mass on the end of a rigid rod. The task was to grasp the object by the handle at the base of the rod and rotate it back and forth between visual targets. Subjects were told that the object might wobble during the rotation and that they should try to maintain the handle as still as possible within the central home region. We ensured that the wrist operated near the midpoint of its range of motion so that a comfortable posture was adopted. Subjects maintained this posture throughout the experiment.

The visual feedback was provided by a projection system that overlaid a visual image in the plane of the movement as previously described [33]. The visual object (Figure 1B) consisted of a circular handle (radius 0.5 cm) attached to a 4 cm square mass by an 8 cm rod (width 0.2 cm). The position and orientation of the object was determined by the position and orientation the WristBOT handle. The home region was a 1 cm radius disc and the start and end targets for rotation were oriented rectangles (0.6 by 2.5 cm) continuous with the disc (Figure 1B). The orientation of the object could be varied between trials. For a given object orientation, subjects performed trials that alternated between clockwise (CW) and counter-clockwise (CCW) rotations of amplitude 40° between the two targets. The targets were ±20° relative to the central orientation at which the object was said to be presented. For example, when the object was presented at −90° the CW and CCW targets were at −70° and −110°, respectively. A trial began with the handle of the object stationary within the home region and the rod of the object aligned with either the CCW target or the CW target. The movement was cued by a tone and the appearance of the second target. The trial ended when subjects had rotated the object to reach the second target. Subjects were required to make the movement within 400 ms. They were warned if they took longer and had to repeat the trial if the movement exceeded 500 ms. Rest breaks (30–60 s) were given every 3–5 minutes.

### Object dynamics

The WristBOT simulated the dynamics of an object (Figure 1B) that consisted of a point mass (*m*) at the end of a rigid rod (length *r*). In all experiments the object had a rod length of 8 cm and, except were explicitly stated, the mass was equal to 1% of the subject's body mass (1% BM). When rotating the object, subjects experienced a torque (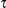) that depended on the angular acceleration (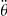) of the handle:
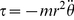
(1)


Subjects also experienced a force at the handle that consisted of two orthogonal components. The first component was associated with the tangential force (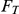), which depended on the angular acceleration of the handle:
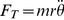
(2)


Note that 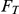 acts in the direction of the tangential acceleration of the mass, and is thus perpendicular to the rod. The second component was associated with the centripetal force (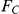), which depended on the angular velocity (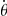) of the handle:
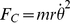
(3)


Note that 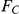 acts perpendicular to the tangential velocity of the mass (towards the centre of rotation), and is thus parallel to the rod.

The resultant force vector (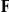) experienced by the subject at the handle is given by:
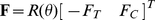
(4)where 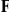 is the two-dimensional force vector (in the coordinate system of the WristBOT, as specified in Figure 1B), 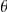 is the angle of the rod (0° is aligned with the *y*-axis) and 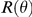 is a 2×2 clockwise rotation matrix.

To avoid the need to compute angular velocity and acceleration, the dynamics were approximated by a simulation in which the point mass was attached to the end of the rod by a stiff spring (3000 N/m). Translation and rotation of the object caused the spring to stretch, which then generated forces (and torques) on the handle. At the same time, these forces were used to update the state of the simulated mass. A small amount of damping was applied to the mass to prevent oscillations (7 N m^−1^ s). An analysis of the kinematics and the forces and torques generated by the WristBOT during the task verified that this approximation accurately captured the dynamics of the object (see Figure S1 in Text S1).

### Experiments

A specific description of the individual experiments can be found in the following sections. In general, each experiment consisted of multiple trials in which the visual orientation and dynamics of the object could be varied from trial to trial. Subjects always experienced the torque associated with rotating the object on every trial, whereas the forces experienced could be varied across trials in three different ways. On “exposure” trials, subjects experienced the full dynamics of the object such that the manipulandum produced the forces associated with rotating the object. On these trials, movement of the handle during the rotation was caused by the sum of the forces produced by the object and the forces produced by the subject. Specifically, if subjects produced forces that exactly opposed those produced by the object, the handle would remain stationary during the rotation (as per the task requirements). On “zero-force” trials, the forces associated with rotating the object were turned off and the manipulandum produced no forces. On these trials the handle was free to move and any forces produced by subjects as they rotated the object resulted in a displacement of the handle. Finally, on “error-clamp” trials, the manipulandum simulated a stiff two-dimensional spring (1000 N/m) centered on the handle position at the start of the trial. On these trials, any forces produced by the subject as they rotated the object were recorded as equal but opposite forces generated by the spring. These error-clamp trials minimize kinematic errors [34] and thus minimize adaptation (or de-adaptation), allowing the anticipatory forces produced by subjects as they rotate the object to be assessed.

### Data collection and statistical analysis

The position and orientation of the handle and the force and torque generated by the manipulandum were saved at 1000 Hz for offline analysis using Matlab (R14, The MathWorks Inc., Natick, MA, USA). Two measures were used to characterize the trial-by-trial performance of the subjects during the task. On zero-force and exposure trials, the peak displacement of the handle was measured, relative to its position at the start of the trial. The peak displacement of the handle (in cm) is a measure of error, because the task required subjects to keep the base of the object as still as possible during the rotation. A peak displacement of zero would thus indicate perfect performance. On error-clamp trials, the forces produced by subjects were measured. Subjects produce these forces in order to oppose the perturbing forces generated by the object. The peak force can be regarded as a measure of the subject's estimate of the mass of the object. As such, for a given error-clamp trial, we divide the peak force produced by the subject by the peak force which would have been generated by the object. We refer to this dimensionless ratio as the adaptation, which has a value of 1 if subjects produce forces which exactly compensate for the mass of the object. All statistical tests were performed using Matlab. All t-tests were paired and two-tailed.

### Modeling

Various state-space models have been recently proposed to explain adaptation to dynamic [15], [16], [17], [20] and kinematic (visuomotor) perturbations [18], [19]. These models have yet to be applied to object manipulation, especially in light of experimental results that characterize trial-by-trial adaptation to object dynamics [29], [30], [32].

In the simplest case, the state-space adaptation model takes the form of a single-rate (single-state) model (SRM) as follows:
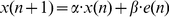
(5)where *x*(*n*) is the state of the model on trial *n*, *α* is the retention constant, *β* is the learning-rate constant, and *e* is the error given by:
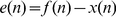
(6)


In the case of dynamic and visuomotor perturbation experiments, *f* is typically taken to be a dimensionless value which represents the magnitude and sign of the external perturbation [17], [19]. In our case, *f* is the mass of the object. For simplicity we consider *f*, *x* and *e* in Equation 5 and 6 to be dimensionless quantities, where *x* can be thought of as the subject's internal estimate of the mass and *e* can be thought of as the error in this estimate. Where reported, values of *x* in the model are referred to as adaptation. To facilitate comparison with the equivalently named experimental quantity described above, adaptation in the model is expressed as the ratio of the estimated mass (*x*) to the actual mass (*f*). In all experiments, *f* was set to the experimental mass of the object, which (as described above) was 1% BM in all experiments, except where explicitly stated.

Recently, a dual-rate (dual-state) model (DRM) has been successfully applied to the results from both dynamic [17] and visuomotor [19] perturbations:
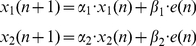
(7)


The rate-specific states sum together to produce the net output state of the model:
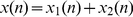
(8)


In these previous studies, the rate-specific states and the relative values for their retention and learning-rate constants have been associated with fast and slow adaptation processes [17], [19]. In the current study, we fit both the SRM and DRM to the results of Experiment 1 (details in the following section) in order to determine whether adaptation to familiar object dynamics is mediated by a single-rate or dual-rate process.

 The single-rate and dual-rate models described above are context-independent models. To explain context-specific adaptation, multiple-state context-dependent versions of these models have been proposed [15], [19]. The multiple-context form of the single-rate model (MCSRM) in the current study is given as follows:
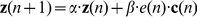
(9)where **z**(*n*) is a vector of the context-specific states on trial *n* and **c** is the context-selection vector (described below). The net output state of the model is the sum of the context-specific states, weighted by the context-selection vector:
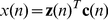
(10)where *x*(*n*) is the net output state of the model on trial *n*.

 As originally described by Lee and Schweighofer [19], the context-selection vector **c** defines which context is active on a given trial. It does this in a binary manner. Specifically, the element in **c** associated with the current context is 1 and all other elements are 0. In this previous study, the context was the direction of movement in a visuomotor perturbation task. In the current study, the context is the visual orientation of the object specified in increments of 22.5°. As such, the context-dependent state vector **z** and the context-selection vector **c** contain 16 elements (covering 360° in 22.5° steps).

In our previous study, we reported a Gaussian-tuned pattern of generalization across different visual orientations of the object [32]. This graded pattern of generalization cannot be reproduced by the binary context-selection vector from the Lee and Schweighofer model, described above. Rather, we use a Gaussian function tuned to the visual orientation of the object to specify the 16 elements of the context-selection vector. The shape of the function (Figure 1D) is defined by two parameters which specify the standard deviation of the Gaussian (*σ*) and its offset (*d*). The function is normalized to be 1 at the current orientation *θ*(*n*) and decays to *d* at *θ*(*n*)±180°. The context-selection vector (**c**(*n*) in Equations 9 and 10) thus becomes **c**(*θ*(*n*),*σ*,*d*), which is given by:
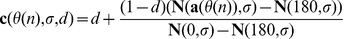
(11)


The function **N**(*x*,*σ*) in Equation 11 is a zero-mean Gaussian, as follows:
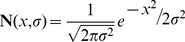
(12)


The function **a**(*θ*) in Equation 11 specifies a 16 element vector (adjusted to the circular range of ±180°) which centers the tuning function at the current orientation, as follows:
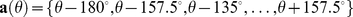
(13)


The MCSRM with the Gaussian context-selection function described above has 4 parameters (MCSRM4). In order to test our assumption that the context-selection function was Gaussian in form, we also considered a model in which the individual elements of **c** were free parameters. Assuming symmetry and a fixed value of 1 at the current orientation, 8 parameters defined the context-selection vector in this version of the model to give a total of 10 parameters (MCSRM10).

In the context-independent versions of the model (SRM and DRM, Equations 5 to 8), the object is experienced at a single orientation and the error (*e*) is calculated simply as the difference between the actual mass of the object (*f*) and the subject's estimate of the mass (*x*). However, in the context-dependent versions of the model (MCSRM4 and MCSRM10, Equations 9 and 10), the object can be experienced at multiple orientations. This complicates the calculation of error because the displacement of the object during the task will be influenced by the compliance of the arm, which varies for perturbations in different directions [35], [36]. To account for this, the calculation of error in the context-dependent versions of the model includes a compliance term which was determined experimentally. Specifically, we define a compliance-dependent error function for the model as follows:
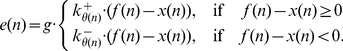
(14)


As in the original error function (Equation 6), the error is due to the difference between the actual mass of the object (*f*) and the subject's estimate of the mass (*x*). In this case, however, the error is the product of this difference with the compliance factor (*k*) and the gain factor (*g*). The subscripts and superscripts on *k* in Equation 14 allow the compliance to vary for different orientations of the object (*θ*) and for positive and negative errors, respectively (*k^+^* when *f*>*x*, *k^−^* when *f*<*x*). This latter feature of the function allows the compliance to be different during adaptation and de-adaptation. Specifically, during adaptation, displacement of the hand is due to the object producing net forces on the subject. In contrast, during de-adaptation, displacement of the hand is due to the subject producing net forces on the object. The compliance can be different in each case.

The value of *k* for a range of orientations was determined experimentally in a separate group of subjects (see Experiment S1 in Text S1 for full details). It then remained fixed for all other experiments and subject groups. Because the peak displacement for these different subject groups could vary over a small but critical range, the gain factor (*g*) in Equation 14 was included as a free parameter in those models which implemented the compliance function. This parameter was close to 1 in all cases (range 0.86 to 1.14). As for peak displacement in the experiments, the compliance-dependent error has units cm.

When fitting the various models to experimental data, parameters were estimated by a non-linear least-squares procedure performed in Matlab (lsqnonlin). The absolute error output of the model (from either Equation 6 or 14) was fit to the peak displacement trials series for each experiment. The mean peak displacement across subjects was used because the data for individual subjects was too noisy to obtain reliable fits. Confidence intervals for parameter estimates were calculated using a boot-strap procedure [17]. Specifically, the boot-strap was performed using 1,000 unique combinations drawn with replacement from the subject pool for each experiment. The model was fit separately to the mean peak displacement trial series for each of the 1,000 unique combinations of subjects. The 95% confidence intervals were calculated as the 2.5 and 97.5 percentile values from the distribution for each parameter obtained across the 1,000 individual fits.

In the case where more than one model was fit to the experimental data, model selection was performed using the Bayesian Information Criterion (BIC). The BIC for a particular model combines a “reward” for the goodness of fit with a “penalty” for the number of free parameters:
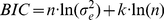
(15)where *σ^2^_e_* is the variance in the residual errors of the fit, *k* is the number of free parameters and *n* is the number of data points (the number of trials). Taking the difference in BIC values for two competing models approximates half the log of the Bayes factor [37]. A BIC difference of greater than 4.6 (a Bayes factor of greater than 10) is considered to provide strong evidence in favor of the model with the lower BIC value [38].

### Experiment 1 – Single-context adaptation and de-adaptation

**Figure 2:**
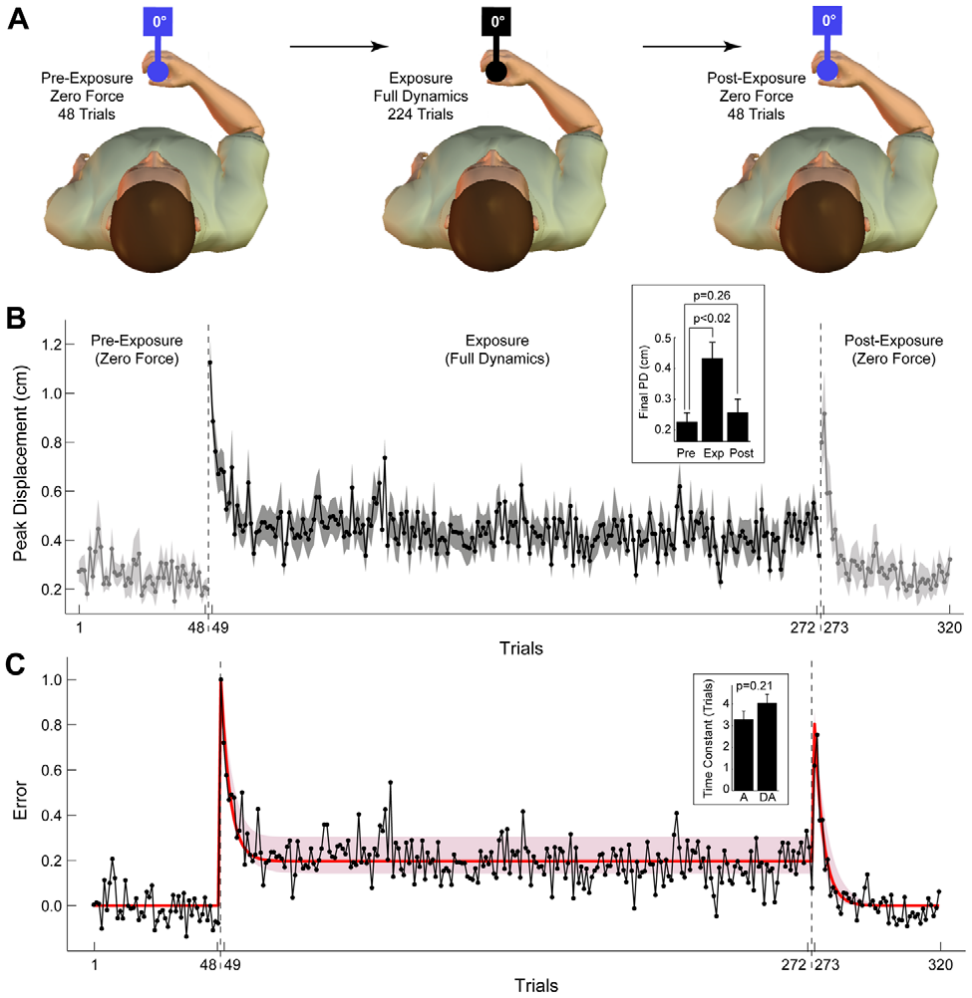
Single-context adaptation and de-adaptation (Experiment 1), experimental results and model fit. **A** Subjects (n = 8) experienced a pre-exposure phase of 48 zero-force trials (left), followed by an exposure phase of 224 trials with full object dynamics (middle), followed by a post-exposure phase of 48 zero-force trials (right). The object was presented at 0° on all trials. **B** The peak displacement trial series for the experiment. Data points are means across subjects and shaded area are standard errors. Initial and final light-grey shaded plots are pre-exposure and post-exposure phases, respectively. Dark-grey shaded plot is exposure phase. Inset shows final peak displacement (PD) for each phase (final 8 trials for each phase; mean and standard error across subjects; p-values are for two-tailed paired t-tests as indicated). **C** The normalized peak displacement trial series for the experiment and the model fit. Black trace is mean across subjects from the experiment. Red line is the fit for the single-rate model (SRM). Pink shading shows 95% confidence limits of the fit (see text for details). Inset shows the exponential time constants (mean and standard error across subjects) for adaptation (during exposure) and de-adaptation (during post-exposure; p-value is for a two-tailed paired t-test).

The first experiment was designed to examine adaptation and de-adaptation in a single context (Figure 2A). Subjects (n = 8) were presented with the object at 0°. They performed an initial familiarization block of 48 pre-exposure trials during which the forces associated with the dynamics of the object were turned off (zero-force trials). The main experiment consisted of 320 trials and began with a second block of 48 pre-exposure (zero-force) trials. Subjects then experienced the full dynamics of the object (both torques and the perturbing forces of the object) during an exposure phase of 224 trials. In the final 128 trials of the exposure phase, one error-clamp trial was inserted randomly every 8 trials for a total of 16 error-clamp trials (8 CW and 8 CCW trials). During the final post-exposure phase of 48 trials, the object forces were again turned off (zero-force trials). Subjects were given rest breaks, randomly timed to occur every 3–5 minutes.

We fit both the single-rate and the dual-rate context-independent models (SRM and DRM) to the trial series of the normalized mean peak displacement from Experiment 1. Specifically, the mean peak displacement was calculated for each trial across subjects and then normalized across trials so that the mean of the pre-exposure phase was 0 and the maximum error across all trials was 1.

To characterize the time constant of adaptation (during the exposure phase) and de-adaptation (during the post-exposure phase), we fit a single exponential function to each individual subject's data:
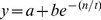
(16)where *n* is the trial number. We calculated the mean time constant (*t*) across subjects.

### Experiment 2 – Multiple-context de-adaptation

**Figure 3:**
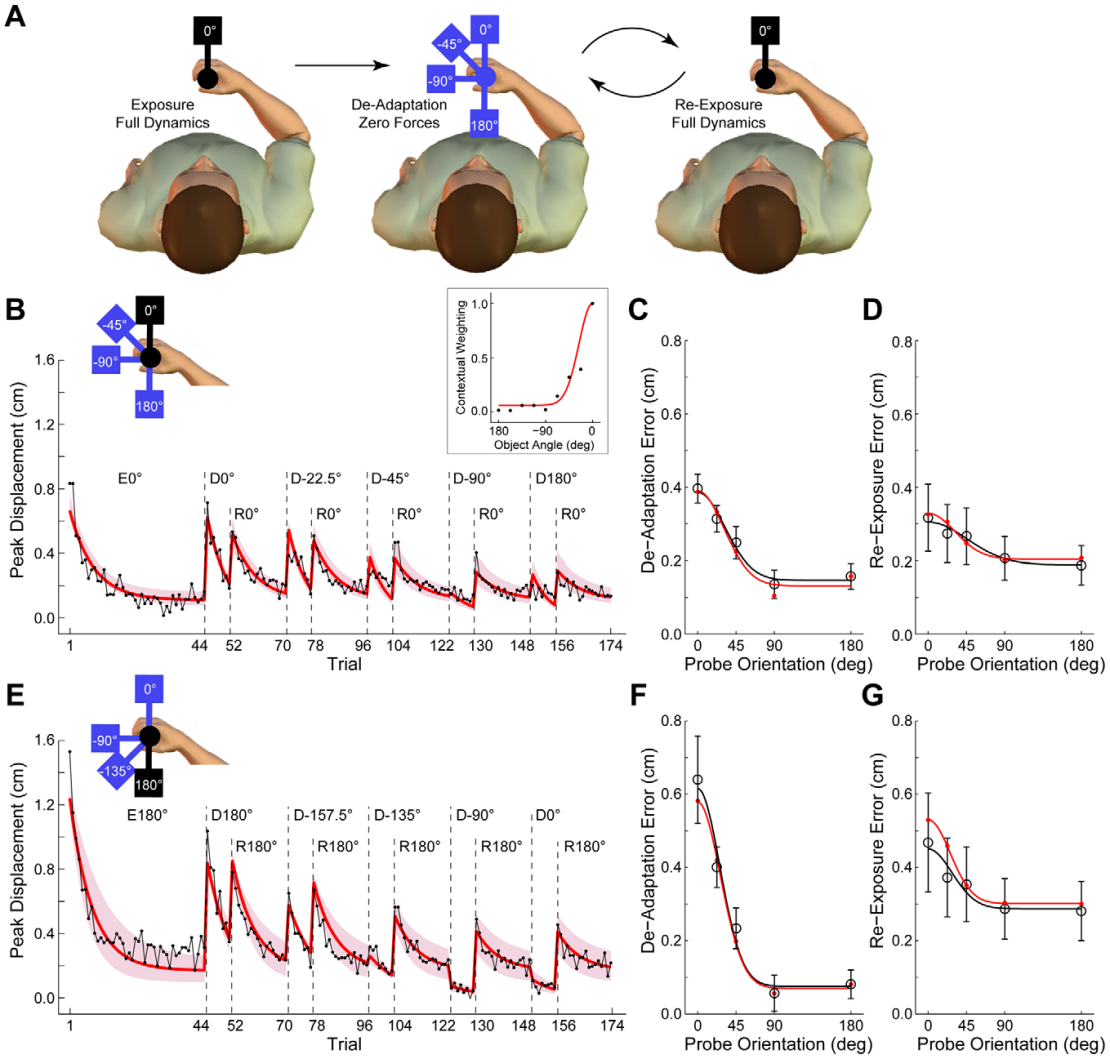
Multiple-context de-adaptation (Experiment 2), experimental results and model fit. **A** Subjects (n = 12 in each group) were initially exposed to the full dynamics of the object at a training orientation of 0° (group 1; left panel) or 180° (group 2; not shown). They then experienced multiple blocks of 30 trials in which they were first partially de-adapted with 8 zero-force trials presented at 1 of 5 probe orientations (0, 22.5, 45, 90 and 180° relative to the training orientation; middle panel; object at 22.5° removed for clarity) and then re-exposed (full dynamics) at the training orientation for 18 trials (right panel). **B** The peak displacement trial series for the experiment (black trace) and the model fit (red trace with pink shading showing 95% confidence limits of the model fit) for 0° training group. Eθ° and Rθ° are exposure and re-exposure trials at the training orientation (0°), respectively. Dθ° are zero-force de-adaptation blocks at 1 of 5 probe orientations. Subjects performed 3 repeats for each probe orientation in a random sequence (3×5 = 15 de-adaptation blocks). Experimental data is a composite in which the 3 repeats for each probe orientation are averaged and sorted in order of relative orientation (see text for full details). **C** De-adaptation error (mean peak displacement over first 4 de-adaptation trials) for each probe orientation for 0° training group. Black symbols are means and standard error across subjects. Red symbols are equivalent analysis of model fit. Black and red lines show a half-Gaussian fit to experimental and model points, respectively. **D** Re-exposure error (mean peak displacement over first 4 re-exposure trials) for each probe orientation for 0° training group, plotted as in panel C. **E** Peak displacement trials series for the experiment and model fit for 180° training group, plotted as in panel B. **F** De-adaptation error for 180° training group, plotted as in panel C. **G** Re-exposure error for 180° training group, plotted as in panel D.

The second experiment was designed to examine the context-dependent pattern of generalization across multiple object orientations after exposure to the dynamics of the object at a single orientation (Figure 3A). Two groups of subjects (n = 12 in each group) were first exposed to the object at 0° (group 1) or 180° (group 2) for 64 trials. They were then presented with multiple blocks of 30 trials in which they were first partially de-adapted with 8 zero-force trials presented at one of five possible probe orientations (group 1: 0, −22.5, −45, −90 and 180°; group 2: 180, −157.5, −135, −90 and 0°). They were then re-exposed to the full dynamics of the object for 18 trials at the training orientation. The first 2 trials immediately before and immediately following the zero-force de-adaptation trials were error-clamp trials presented at the training orientation, during which the forces produced by subjects were measured. Probe orientations were presented in a pseudo-random order such that each probe orientation was presented once per cycle, with subjects performing 3 cycles (3 cycles×5 blocks per cycle = 15 blocks). The peak displacement of the handle during the first 4 de-adaptation trials was used as a measure of the magnitude of the anticipatory forces produced by subjects at each probe orientation. In addition, the peak displacement during the first 4 re-exposure trials (at the training orientation), was also measured. Note that the probe orientations for each group (0° and 180°) represented identical steps relative to the training orientation (0, 22.5, 45, 90 and 180°). Rest breaks were given every 3–5 minutes as in Experiment 1.

We fit the 4 parameter and 10 parameter versions of the single-rate multiple-context model (MCSRM4 and MCSRM10, respectively) to the trial series of the mean peak displacement across subjects. Both functions implemented the compliance-dependent error function (Equation 14, described above). The 4 parameter version of the MCSRM included the retention and learning-rate constants (*α* and *β*) as well as the width (*σ*) and offset (*d*) parameters for the Gaussian context-selection function. The 10 parameter version of the model included the 2 rate constants as well as 8 values specifying the individual elements of the context-selection vector, as described above.

### Experiment S1 – Estimating the compliance function

The compliance factor in the compliance-dependent error function (*k* in Equation 14) was estimated by exposing subjects to the object at multiple orientations (see Experiment S1 in Text S1 for full details). Briefly, subjects (n = 12) experienced the object at 5 different orientations (0, −45, −90, −135 and 180°). They were adapted and then de-adapted to the object dynamics multiple times at each orientation. A modified version of the SRM was then fit to the mean peak displacement trial series. In this modified SRM, the original error function (Equation 6) was replaced by the compliance-dependent error function (Equation 14). The model fit the *α* and *β* parameters of the SRM (Equation 6) along with 10 parameters which defined the compliance factor *k*.

### Experiment 3 – Adaptation to different masses

**Figure 4:**
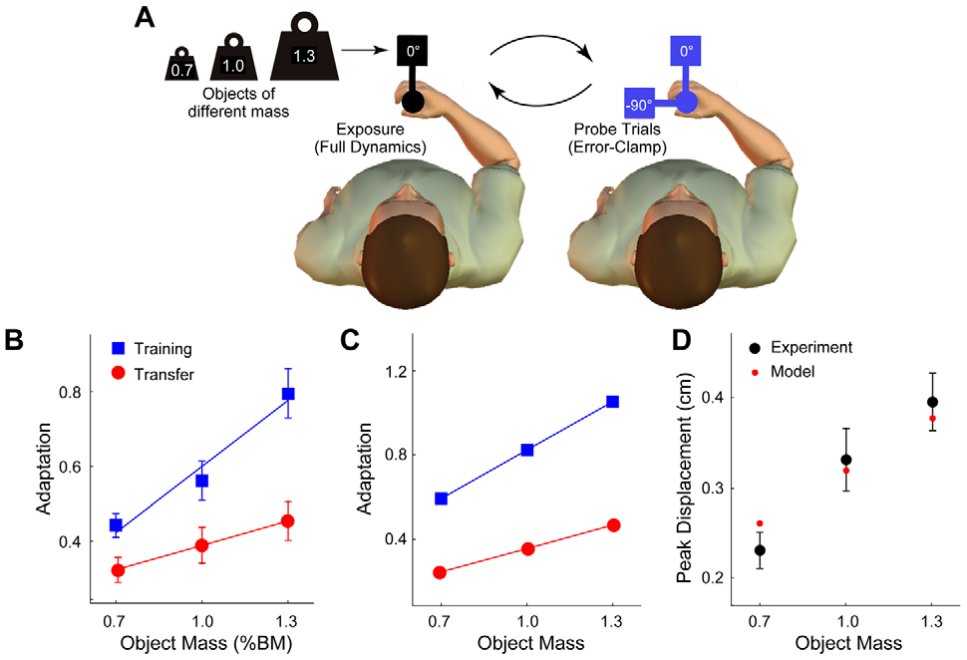
Adaptation to objects of increasing mass (Experiment 3), experimental results and model predictions. **A** Subjects (n = 8) were exposed to an object with 3 different masses in blocks of 90 trials. They first experienced the object at the training orientation (60 trials at 0°; left panel) and were then presented with random error-clamp trials (right panel) at the training orientation (3 error-clamp trials at 0°) or a novel transfer orientation (3 error-clamp trials at −90°). The 6 error-clamp trials were randomly inserted into a block of 30 exposure trials (1 every 5 trials). Subjects experienced the 3 masses in a pseudo-random order. **B** Adaptation in the experiment as a function of increasing object mass for training (blue squares) and transfer (red circles) orientations of the object (means and standard errors across subjects). Adaptation was measured using error-clamp trials and is expressed relative to the 1.0% BM object (see main text). The lines show the mean of the individual linear fits to each subject for the training (blue) and transfer (red) orientations. **C** Adaptation in the model as a function of the object mass, plotted as in panel B. The model simulation is for the MCSRM4 with the best-fit parameters from Experiment 2. **D** Final peak displacement from the experimental data (black points show means and standard errors across subjects) and predictions from the model (red points). The model correctly predicted that subjects would tolerate larger errors for objects of larger mass.

The third experiment was designed to examine adaptation to objects of different mass (Figure 4A). Specifically, subjects (n = 8) were exposed to 3 object masses (0.7%, 1.0% and 1.3% of the subject's body mass). The object was presented in separate blocks of 90 trials for each mass (in pseudo-randomized order). Each block began with an exposure phase of 60 trials during which subjects experienced the object at the training orientation of 0°. In the subsequent 30 trials, one error-clamp trial was inserted randomly every 5 trials. For these error-clamp trials, the object was presented at 0° (the training orientation) or at −90° (the transfer orientation). By examining the forces generated by the subjects on error-clamp trials we could assess their adaptation to the particular mass of the object at the training orientation and at the novel probe (transfer) orientation. Rest breaks were given every 3–5 minutes as in Experiment 1. Simulated results were generated for this experiment using the selected MCSRM and its best-fit parameters from Experiment 2.

### Experiment 4 – Dual-context adaptation

**Figure 5:**
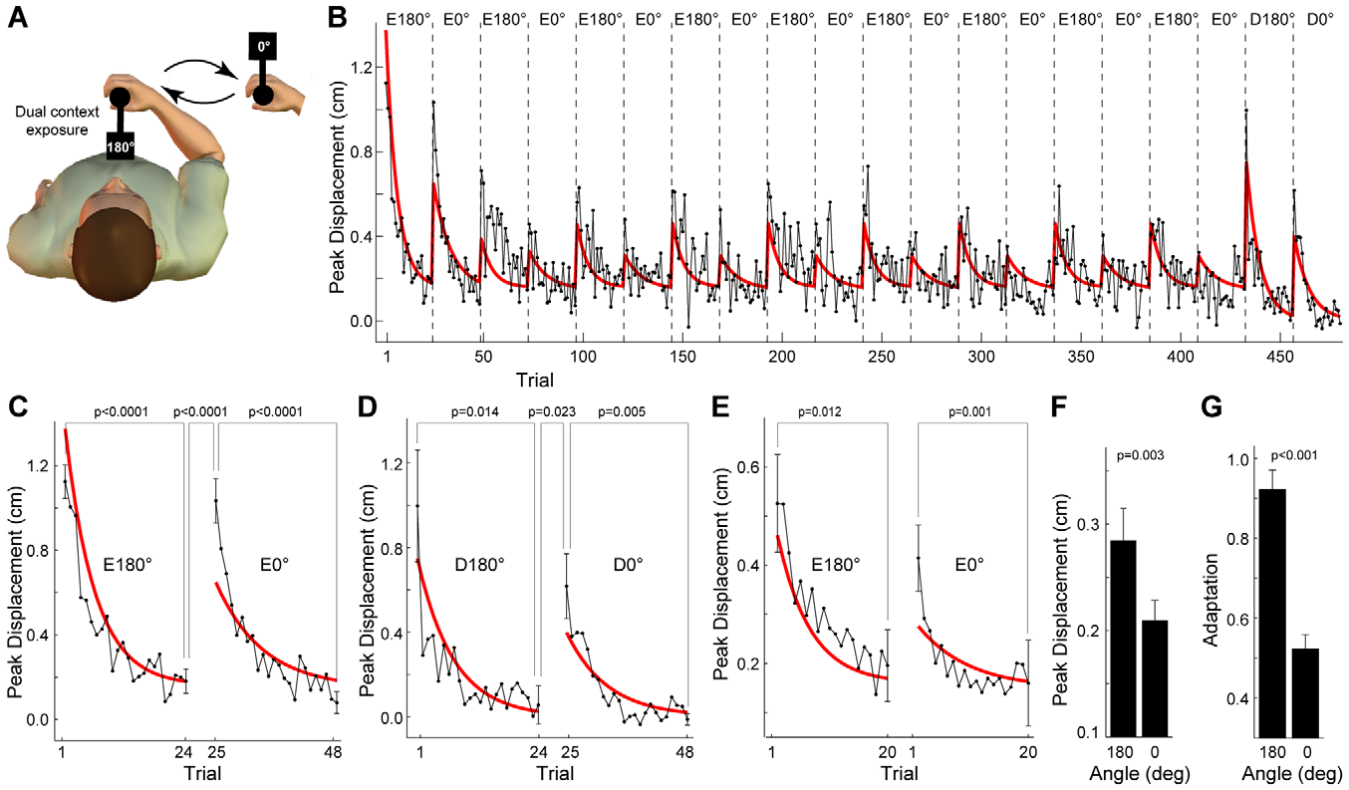
Dual-context adaptation (Experiment 4), experimental results and model predictions. **A** Subjects (n = 8) experienced the object at two orientations (180° and 0°) which alternated in blocks of 24 trials (4 random error-clamp catch trials and 20 exposure trials under full dynamics). **B** The peak displacement trial series for the experiment (black trace) and model prediction (red trace). The model prediction is for the MCSRM4 with the best-fit parameters from Experiment 2. Eθ° and Dθ° are exposure and de-adaptation blocks (respectively) at each orientation. **C** Experimental (black trace) and model (red trace) peak displacement trial series for the initial exposure blocks at 180° and 0°. Error bars on first and last trial in each block are standard errors. P-values are for two-tailed paired t-tests, as indicated. **D** Experimental and model peak displacement trial series for the de-adaptation blocks at 180° and 0°, plotted as in panel C. **E** Experiment and model peak displacement trial series averaged over blocks for each orientation (not including the initial adaptation blocks), plotted as in C. **F** Experimental peak displacement averaged across blocks for each orientation in the final half of the experiment. P-value is for a two-tailed paired t-test. **G** Experimental adaptation measured using error-clamp trials for each orientation. P-value is for a two-tailed paired t-test.

The fourth experiment was designed to examine adaptation when the object alternated sequentially between two different contexts (Figure 5A). Subjects (n = 8) performed blocks of 24 trials with the object presented at 180° and 0° consecutively across pairs of blocks. A cycle thus consisted of a pair of blocks at 180° and 0°. An initial pre-exposure cycle and a final post-exposure cycle were performed (2 blocks×24 trials per block = 48 trials). These consisted of zero-force trials as in Experiment 1. The exposure phase included 9 cycles (18 blocks for a total of 432 trials). The entire experiment consisted of 11 cycles (11 cycles×2 blocks per cycle×24 trials per block = 528 trials). Rest breaks were given every 3–5 minutes as in Experiment 1. Predictions for this experiment were generated using the selected MCSRM and its best-fit parameters from Experiment 2.

### Experiment 5 – Adaptation to multiple contexts

**Figure 6:**
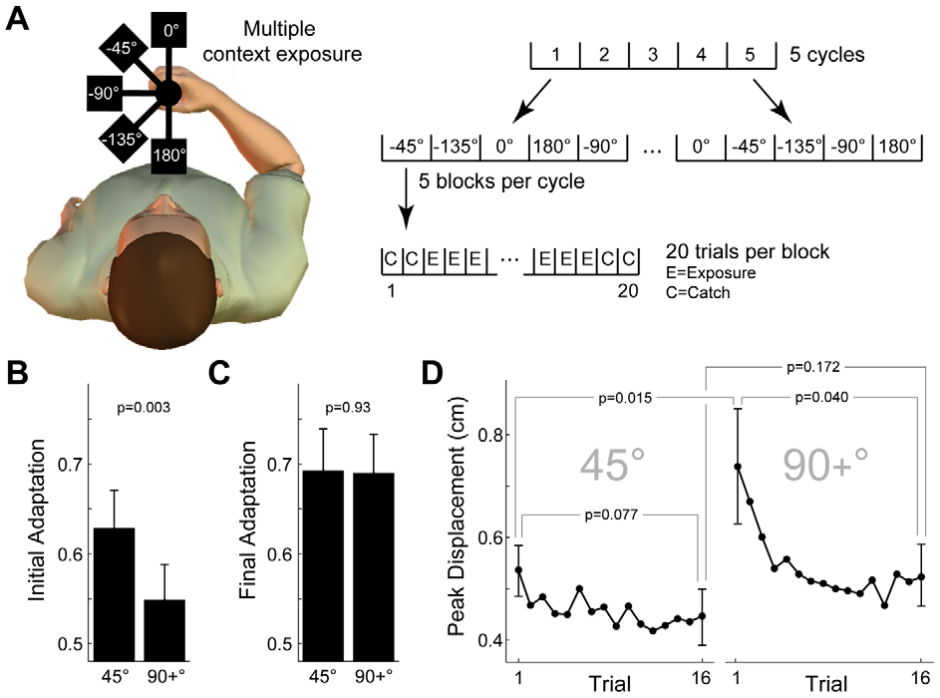
Multiple-context adaptation (Experiment 6), experimental results and model predictions. **A** Subjects (n = 8) were exposed to the full dynamics of the object at multiple orientations (0°, −45°, −90°, −135°, 180°), with each orientation presented in multiple blocks of 20 trials. Blocks were grouped into cycles (5 blocks per cycle consisting of 5×20 = 100 trials) such that each orientation was presented once per cycle (in pseudo-random order). Subjects performed 5 cycles (5×100 = 500 trials). The first 2 trials and last 2 trials of each block were error-clamp trials (C). The remaining 16 trials were exposure trials (E) under the full dynamics. **B** Experimental results for the initial adaptation (measured using error-clamp trials). The 45° bin includes only absolute changes in orientation of 45° whereas the 90+° bin includes absolute changes in orientation of 90° or larger. P-value is for a two-tailed paired t-test. **C** Experimental results for the final adaptation, plotted as in panel B. **D** The peak displacement trial series across the 16 exposure trials for blocks in the 45° and 90+° bins (mean across subjects; error bars on first and last trials of each block are standard error). P-values are for two-tailed paired t-tests as indicated.

The fifth experiment was similar to Experiment 4 and was also designed to examine adaptation when the object switched between different contexts (Figure 6A). In this case, the object was presented at five different orientations. Subjects (n = 8) performed multiple exposure cycles that consisted of blocks of 20 trials in which the object was presented at one of five orientations (0°, −45°, −90°, −135°, 180°). The first two and the last two trials of each block were error-clamp trials. The remaining 16 trials were under the full dynamics of the object. A cycle consisted of a sequence of 5 blocks with each orientation presented once (in a pseudo-random order). Subjects performed two pre-exposure cycles (error-clamp trials but only 4 trials: 2 cycles×5 blocks×4 trials = 40 trials), five exposure cycles (5 cycles×5 blocks×20 trials = 500 trials) and two post-exposure cycles (as in the pre-exposure). This gave a total of 580 trials. Rest breaks were given every 3–5 minutes as in Experiment 1. Predictions for this experiment were generated using the selected MCSRM and its best-fit parameters from Experiment 2.

The model and experimental results for Experiment 5 were analyzed as follows. Values for the two measures (peak displacement and adaptation) were binned for each block based on the absolute change in orientation relative to the previous block. The analysis included two bins; one for absolute changes in orientation of 45° and another for absolute changes of 90° or larger (denoted 90+°). The 90+° bin was chosen because relative changes in orientation greater than 90° did not occur often enough to allow separate bins. Data from the first exposure cycle was not included because it represented the initial adaptation to the object dynamics.

### Previous object manipulation studies

Two previous studies of object manipulation are relevant to the model presented in the current study. These previous studies examined grip force adaptation during a bimanual object manipulation task. Specifically, they characterized the time-course of adaptation and de-adaptation of grip force [30] and the pattern of generalization following single-context and dual-context exposure [31]. The MCSRM was fit concurrently to data from these four experiments (see Figure S6 and further details in Text S1).

## Results

Subjects rotated a virtual object by grasping and rotating the handle of the WristBOT robotic manipulandum (Figure 1A) [32], [33]. The WristBOT produced the forces and torques associated with the dynamics of the object (Figure 1B). Real-time visual feedback of the object was projected over the subject's hand (Figure 1C). On pairs of trials, subjects rotated the object CW and CCW through 40° between two visually presented targets. The orientation of the object and targets was varied in order to present the object at different orientations (inset, Figure 1C). Subjects were asked to keep the handle stationary within the home position. This required them to produce forces to stabilize the object as they rotated it. Performance was measured as the peak displacement of the handle during the rotation and error-clamp trials were used to measure the anticipatory forces produced by subjects (see Methods for full details).

### Experiment 1 – Single-context adaptation and de-adaptation

The first experiment examined adaptation and de-adaptation of subjects (n = 8) to the dynamics of the object at a single orientation (Figure 2A). During the initial pre-exposure (zero-force) phase of the experiment, displacements of the handle were small (initial light grey shaded plot in Figure 2B). The mean peak displacement across the final 8 trials of the pre-exposure phase was 0.23±0.08 cm (subject mean ± standard deviation). In the exposure phase (dark grey shaded plot in Figure 2B), upon introduction of the forces associated with the rotational dynamics of the object, displacement increased markedly on the first exposure trial to 1.13±0.24 cm, falling rapidly over subsequent trials. The mean peak displacement across the final 8 trials of the exposure phase was 0.43±0.15 cm, significantly larger than the final peak displacement for the pre-exposure phase (two-tailed paired t-test, p<0.02; see inset of Figure 2B). During the post-exposure phase, when the object forces were again turned off, displacement increased on the first post-exposure trial to 0.80±0.31 cm, falling rapidly over subsequent trials (final light grey shaded plot in Figure 2B). The mean peak displacement across the final 8 trials of the post-exposure phase was 0.26±0.13 cm, which was not significantly different from the final peak displacement for the pre-exposure phase (two tailed paired t-test, p = 0.26; see inset of Figure 2B).

### Single-context adaptation and de-adaptation – Model fitting

The mean peak displacement across subjects was normalized for model fitting by subtracting the mean displacement across the final 8 trials of the pre-exposure phase and dividing by the maximum displacement across all trials. Peak displacement data normalized in this way for model fitting is referred to simply as “error” (black trace in Figure 2C).

Two alternative models were fit to the single-context experiment. The first was the context-independent single-rate model (SRM) described in Equation 5, which has two free parameters. The second was the context-independent dual-rate model (DRM) described in Equations 7 and 8, which has four free parameters. The SRM and DRM are equivalent to the single-state single-rate model and two-state multi-rate model of Smith et al [17], respectively. Model parameters were estimated by a least squares minimization function in Matlab. Model selection was performed using BIC (see Methods and Equation 15).

Both models were able to reproduce the time-course of adaptation and de-adaptation in the experimental data (Figure 2C shows the SRM fit; Figure S3-A in Text S1 shows the fit for both SRM and DRM). The best-fit parameters for the SRM were *α* = 0.9513 and *β* = 0.2150 (*R^2^* = 0.7131). The best-fit parameters for the DRM were *α_1_* = 0.9808, *β_1_* = 0.0139, *α_2_* = 0.9453, *β_2_* = 0.2053 (*R^2^* = 0.7153). The difference in BIC values for the two models was 8.3, providing strong evidence in favor of the SRM (see Methods). Thus, despite the slightly better fit to the data achieved by the DRM, its additional parameters were not justified. The 95% confidence limits for the SRM parameters were *α* = 0.9090–0.9796 and *β* = 0.1553–0.2668 (estimated using a boot-strap procedure, see Methods). The 95% confidence limits for the model fit to the experimental data are shown in Figure 2C (pink shading on the red line).

The BIC analysis, which selected the SRM over the DRM, suggests that adaptation to familiar object dynamics is mediated by a single-rate process. An analysis of the exponential time constants for adaptation and de-adaptation provided further evidence for a single-rate process. Specifically, we fit exponential functions (Equation 16) to the adaptation and de-adaptation phases of Experiment 1 individually for each subject. The mean time constant for adaptation was not significantly different from the mean time constant for de-adaptation (adaptation: 3.3±1.1 trials; de-adaptation: 4.0±1.1 trials; two tailed paired t-test, p = 0.21; see inset of Figure 2C). Thus, we found no evidence for the phenomenon referred to as fast de-adaptation which is characteristic of adaptation to novel dynamics [17], and dual-rate adaptation processes [17].

We found further evidence to support a single-rate process by performing simulations and an extensive search of the DRM parameter space (see Text S1 for full details). First, the DRM parameters obtained from fitting the model to Experiment 1 exhibit neither spontaneous recovery nor savings when the appropriate experiments are simulated (Figure S3 in Text S1). These phenomena are characteristic of dual-rate adaptation processes [17] and their absence is thus consistent with a single-rate process. Second, an analysis of the DRM parameter space shows a wide range of solutions which provide a good fit to Experiment 1 (Figure S4 in Text S1). Such parameter redundancy would be expected if a DRM is fit to data generated by a single-rate process. In contrast, the SRM solutions which provide a good fit to Experiment 1 are confined to a narrow region of the parameter space (inset of Figure S4 in Text S1). Finally, we show that by excluding fast de-adaptation, results from Experiment 1 constrain the best-fit DRM solutions to a single-rate subspace which excludes spontaneous recovery and savings (Figure S5 in Text S1). This is especially striking given the wide range occupied by these solutions in DRM parameter space (compare Figure S4 and Figure S5 in Text S1).

### Experiment 2 – Multiple-context de-adaptation

The second experiment examined context-specific de-adaptation at a range of probe orientations after exposure at a single training orientation (Figure 3A). Subjects initially adapted to the dynamics of the object at 0° (group 1, n = 12) or 180° (group 2, n = 12). They then completed multiple de-adaptation blocks at one of five probe orientations (0°, 22.5°, 45°, 90° and 180° relative to the training orientation). After each de-adaptation block, subjects were re-exposed to the dynamics at the original training orientation. Peak displacement during de-adaptation blocks at the various probe orientations gave a measure of context-dependent transfer. Peak displacement during the subsequent re-exposure blocks gave a measure of the context-dependent de-adaptation associated with each probe orientation.

Analysis of the two error-clamp trials immediately before each de-adaptation block (at the training orientation), shows no difference in adaptation as a function of the probe orientation (ANOVA F(4,55) = 0.084, p = 0.696 for group 1 and F(4,55) = 0.151 p = 0.988 for group 2). Therefore, although each subject experienced the probe orientations in a different sequence, they were in a similar state of adaptation at the start of each de-adaptation block. We therefore felt justified in averaging the peak displacement data for the three de-adaptation blocks at each orientation within and then across subjects for the 2 groups. This allowed us to construct a composite trial series which included a single de-adaptation and re-exposure block for each orientation (see black traces in Figure 3B and E).

Consistent with the previous single-context experiment, during initial exposure to the full object dynamics, subjects adapted rapidly to the perturbing forces of the object (E0° and E180° in Figure 3B and E). During the de-adaptation block at each probe orientation (Dθ°), peak displacement on the initial trials was small for probe orientations that were far removed from the training orientation, increased progressively as probe orientations approached that of the training orientation, and reached a maximum value at the training orientation itself. Moreover, for probe orientations close to or at the training orientation, the initially large errors decreased rapidly across the 8 de-adaptation trials. This orientation-dependence for the initial de-adaptation error can be seen in Figure 3C and F (black symbols are means across the first 4 de-adaptation trials for each orientation). A similar pattern was observed during the re-exposure block (R0° and R180° in Figure 3B and E). As can be seen in Figure 3D and G (black symbols are means across the first 4 re-exposure trials for each orientation), the smallest re-exposure errors occurred after de-adaptation trials at probe orientations far removed from the training orientation, whereas progressively larger errors were observed as the de-adaptation orientation approached the training orientation. As with de-adaptation errors, re-exposure errors fell rapidly as subjects quickly re-adapted to the full dynamics of the object (Figure 3B and E).

### Multiple-context de-adaptation – Model fitting

As reported previously [32], the orientation-dependent behavior described above can be well captured by a half Gaussian centered on the training orientation (see black lines in Figure 3C, D, F and G). This motivated the use of a Gaussian tuning function in the multiple-context version of the model. Specifically, the individual elements of the context-selection vector (**c** in Equations 9 and 10) were set according to a Gaussian function centered on the current orientation of the object (see Figure 1D). This version of the model had 4 parameters (MCSRM4): the two rate constants (*α* and *β*) and two additional parameters which specified the width (*σ*) of the Gaussian tuning function and its offset (*d*). To test our assumption that the tuning function was Gaussian in form, we also fit a version of the model in which the individual elements of the context-selection vector were free parameters. This version of the model had 10 parameters (MCSRM10; see Methods for full details). Model selection was performed using BIC, as described above.

Both models were fit concurrently to the data from both groups (0° and 180°). The best-fit parameters for the MCSRM4 were *α* = 0.9811, *β* = 0.0451, *σ* = 26.3° *d* = 0.09 (*R^2^* = 0.8523; see model fit in Figure 3B and E). Fitting the MCSRM10 yielded similar values for the rate parameters (*α* = 0.9812, *β* = 0.0624) with a small improvement in the fit (*R^2^* = 0.8581). The individual values fit for the context-selection vector are plotted in the inset of Figure 3B (black points) and are shown with the Gaussian tuning function from the MCSRM4 fit for comparison (red line). The difference in BIC values between the two models was 23.8, providing strong evidence in favor of the MCSRM4 (see Methods). As such, allowing the individual elements of the context-selection vector to vary independently provided only a small (and unjustified) improvement in the ability of the model to fit the experimental data. Thus, the assumption that the tuning function is Gaussian in form appears to be valid.

The 95% confidence limits for the MCSRM4 parameters were *α* = 0.9760–0.9870, *β* = 0.0300–0.0540, *σ* = 24.2–30.1° and *d* = 0.08–0.13 (estimated using a boot-strap procedure, see Methods). The 95% confidence limits for the model fits to the experimental data are also shown in Figure 3B and 3E (pink shading on the red line). Note that values for the *β* parameter differ substantially between Experiment 1 (*β* = 0.2150) and Experiment 2 (*β* = 0.0451). This lower value for Experiment 2 is expected because in the MCSRM the multiple states associated with neighboring contexts contribute to adaptation. In contrast, in the SRM a single context-independent state is responsible for adaptation, which results in a larger value for the *β* parameter.

Peak displacement data from the MCSRM4 fit was analyzed in the same way as the experimental data in order to produce plots summarizing the orientation-dependent behavior of the model. Results from this analysis of the model data can be compared with the equivalent analysis of the peak displacement data from the experiment. In both cases, the de-adaptation error (Figure 3C and F) and the re-exposure error (Figure 3D and G) from the model well captured the orientation-dependent behavior seen experimentally (red versus black symbols and lines).

In addition to fitting the MCSRM4 concurrently to the data for both groups of subjects, we also fit the model independently to data from each group. This allowed us to test the generality of the parameters with different groups of subjects who experienced the object across a different range of orientations. Importantly, the parameters were similar when the model was fit separately to data from the two groups. For group 1 (training at 0°) the best-fit parameters were *α* = 0.9798, *β* = 0.0635, *σ* = 24.4° *d* = 0.15 (*R^2^* = 0.8598) and for group 2 (training at 180°) the best-fit parameters were *α* = 0.9817, *β* = 0.0412, *σ* = 25.9° *d* = 0.06 (*R^2^* = 0.8956). This represents an important validation for the model because the peak displacement data varies substantially between the two groups (compare especially Figure 3B and E, and Figure 3C and F).

### Experiment 3 – Adaptation to different masses

The third experiment examined adaptation to objects of varying mass (Figure 4A). In separate blocks, subjects (n = 8) were exposed (training orientation 0°) to the dynamics of objects with three different masses (0.7%, 1.0% and 1.3% of the subject's body mass). Adaptation was then examined using error-clamp trials at the training orientation and a novel transfer orientation (−90°). Because the mass of the object varied in this experiment, the level of adaptation was expressed relative to the 1.0% BM object. Adaptation increased with the mass of the object at both the training orientation (Figure 4B, blue squares) and the transfer orientation (Figure 4B, red circles). Subject-by-subject linear fitting yielded slopes that were significantly different between the training and transfer orientation (0.59±0.17 for training, 0.22±0.15 for transfer; two-tailed paired t-test p<0.005; data previously reported [32]).

### Adaptation to different masses – Model simulation

A simulation of Experiment 3 was performed using the best-fit parameters for the MCSRM4 determined from Experiment 2. The model reproduced the pattern of adaptation seen experimentally (compare Figure 4B and C) and made a prediction regarding the final peak displacement associated with each mass. We confirmed this prediction in a new analysis (Figure 4D). Specifically, the model predicted that the final peak displacement would increase with the mass of the object. This was confirmed statistically in an analysis of the experimental data (ANOVA F(2,21) = 0.0839 p<0.005), showing that subjects tolerate larger errors for heavier objects.

### Single-context adaptation versus multiple-context adaptation

The experiments described thus far have involved exposing subjects to the full dynamics of the object in a single context (a single orientation). Even in experiments 2 and 3, in which the object was presented at multiple orientations, exposure to the full dynamics was restricted to a single training orientation. In the case of exposure at two or more orientations, the MCSRM makes several predictions. These predictions are tested in two new experiments described below.

### Experiment 4 – Dual-context adaptation

In the case of dual-context adaptation, in which the object alternates between two orientations in short blocks (180° and 0°; Figure 5A), the MCSRM4 makes two predictions. First, due to the relatively narrow tuning curve in the model (26.3°), there should be little transfer between orientations separated by more than 60°. Therefore, adaptation during the initial exposure for each context should be largely independent. As such, after first adapting to the dynamics at 180°, subjects should adapt essentially “from scratch” to the dynamics at 0°, with little benefit (transfer) from the exposure at 180°. The model similarly predicts that de-adaptation for each context in the post-exposure phase should be largely independent. As such, after first de-adapting to the dynamics at 180°, subjects will have to de-adapt essentially “from scratch” to the dynamics at 0°. Second, the model has a relatively small retention constant (*α* = 0.9811) which means that non-active contexts should de-adapt quickly. Specifically, after the initial adaptation at both orientations, and as the blocks continue to alternate, there will be some amount of de-adaptation in the non-active context. As such, during subsequent blocks at a particular orientation, there will be a small about of re-adaptation within each block.

The results from the MCSRM4 simulation, using the best-fit parameters from Experiment 2, well matched the peak displacement trial series (mean across subjects; n = 8) for the experimental data (Figure 5B; *R^2^* = 0.5996). ). In some cases, the model over- or under-estimated the experimental peak displacement by a few millimeters (for example, the first few trials in the initial exposure blocks, see Figure 5C). However, the model results are a simulation (not a fit) based on parameters obtained from different groups of subjects. As such, small discrepancies, especially for the large displacements associated with initial exposure, are not unexpected.

With regards to the first prediction, the experiment confirmed that during the initial adaptation there would be little transfer between the initial exposure in the first context and the initial exposure in the second context (see Figure 5C). The largely independent adaptation at each orientation was confirmed statistically by comparing peak displacement values on the first and last trials of each of the initial exposure blocks (E180° and E0° in Figure 5C, p<0.0001 in both cases). In addition, upon transition to the second context, peak displacement increased significantly, consistent with the limited transfer predicted by the model (Figure 5C, E180° to E0° p<0.0001; two-tailed paired t-test). The experiment also confirmed that during the final post-exposure blocks there would be little transfer of de-adaptation between the two contexts (see Figure 5D). The largely independent de-adaptation at each orientation was confirmed statistically by comparing peak displacement values on the first and last trials of each of the post-exposure blocks (Figure 5D, p = 0.0141 for D180°, p = 0.0051 for D0°; two-tailed paired t-tests). In addition, upon transition to the second context, peak displacement increased significantly, consistent with the limited transfer predicted by the model (Figure 5D, D180° to D0° p = 0.0230; two-tailed paired t-test).

With regards to the second prediction, the experiment confirmed that, after the initial two adaptation blocks, there would be a small amount of re-adaptation within each block, due to de-adaptation in the non-active context. The re-adaptation within each block can be seen in the peak displacement trial series (Figure 5B), although individual blocks are noisy. The re-adaptation is best appreciated and statistically confirmed when multiple blocks are averaged for each orientation (Figure 5E). Comparing the first and last trial in the average block trial series for each orientation shows that a significant reduction in error occurred within the block (Figure 5E, p = 0.012 for E180°, p = 0.001 for E0°; two-tailed paired t-tests).

Due to the varying compliance associated with the object at 180° and 0° (see Figure S2 in Text S1), the model made a third prediction regarding the peak displacement and adaptation associated with each orientation. Specifically, the relatively higher compliance associated with the object at 180° should result in a higher peak displacement for 180° blocks than for 0° blocks. This was statistically confirmed when peak displacement was averaged across blocks for each orientation (Figure 5F, p = 0.003). In addition, the model predicts that the higher peak displacement experienced at 180° should drive a larger adaptation to the dynamics of the object at this orientation, relative to 0°. This was similarly confirmed when error-clamp trials were analyzed (Figure 5G, p<0.001).

### Experiment 5 – Multiple-context adaptation

The prediction described above regarding de-adaptation in non-active contexts was investigated further in Experiment 5 which examined the case of multiple-context adaptation (Figure 6A). In this experiment, subjects (n = 8) experienced the object at 5 orientations (0°, −45°, −90°, −135°, 180°), with each orientation presented in blocks of 20 trials. Five cycles of 5 blocks were performed and a block for each orientation was presented once per cycle in pseudo-random order.

The MCSRM4 (using the best-fit parameters from Experiment 2) predicts that the degree of de-adaptation that occurs in a particular non-active context will depend on how far it is removed from the active context. For example, during an exposure block in which the object is presented at −45°, the amount of de-adaptation occurring at 0° and −90° will be less than that occurring at −135° and 180°. This is because for orientations close to the active context, some amount of adaptation occurs (due to spread of adaptation from the active context to its non-active neighbors). This spread of adaptation from the active context offsets the effects of de-adaptation (which occurs equally in all contexts). In contrast, at orientations further removed from the active context, the effects of de-adaptation dominate.

As described in the Methods, the peak displacement and adaptation data from the model and experiment were binned based on the relative change in orientation between consecutive blocks. This yielded two bins (45° and 90+°). The model predicted that the initial adaptation at the start of each block should be larger for the 45° bin than for the 90+° bin, whereas the final adaptation at the end of each block should be the same for the two bins. This prediction was confirmed in the experimental results. Specifically, the initial adaptation (measured using two error-clamp trials at the start of each block) was larger for the 45° bin than for the 90+° bin (Figure 6B; p = 0.003 two-tailed paired t-test), whereas the final adaptation (measured using two error-clamp trials at the end of each block) was the same for the two bins (Figure 6C; p = 0.93).

Model predictions were also confirmed in the experimental peak displacement trial series for the binned blocks (Figure 6D). Specifically, the higher level of adaptation for the 45° bin relative to the 90+° bin was reflected by a lower peak displacement for the 45° bin relative to the 90+° bin (p = 0.015, two-tailed paired t-test between the first trial of the 45° and 90+° bins, as shown in Figure 6D). By the end of the block this difference in peak displacement between the two bins had reduced to non-significant levels (p = 0.172, two-tailed paired t-test between the last trial of the 45° and 90+° bins, as shown in Figure 6D). In additional, the degree of re-adaptation within the binned blocks also confirmed model predictions. Specifically, a small (non-significant) amount of re-adaptation occurred during the block for the 45° bin (p = 0.077, two-tailed paired t-test between the first and last trials of the 45° block, as shown in Figure 6D), whereas a larger (significant) amount of re-adaptation occurred during the block for the 90+° bin (p = 0.040, two-tailed paired t-test between the first and last trials of the 90+° block, as shown in Figure 6D).

The results from the two multiple-context adaptation experiments described above show that, even when subjects receive constant exposure to the object dynamics, non-active contexts de-adapt in a manner consistent with the MCSRM.

### Previous object manipulation studies

The MCSRM4 also successfully captured results from two previous studies of object manipulation (see Figure S6 in Text S1). These studies examined grip force adaptation during a bimanual manipulation task. The model concurrently fit the data from four experiments across the two studies, two which characterized the time-course of adaptation and de-adaptation (Figure S6-B and S6-C in Text S1) [30] and two which characterized the pattern of context-specific generalization (Figure S6-D and S6-E in Text S1) [31]. The best-fit parameters were *α* = 0.8122, *β* = 0.2568, *σ* = 18.2° and *d* = 0.16 (*R^2^* = 0.8419).

In addition, to facilitate comparison across the different object manipulation tasks considered in the current study, exponential functions were fit to two studies which have characterized the time-course of adaptation. The first study was the bimanual task described above [30], which yielded a time constant for grip force adaptation of 0.9 trials. The second study examined adaptation during a task in which subjects lifted an object with an asymmetrically offset centre of mass [29], which yielded a time constant for compensatory torque adaptation of 0.9 trials (see Figure S7 in Text S1).

## Discussion

We have used a context-dependent state-space model to examine how subjects adapt when manipulating objects with familiar dynamics. The model reproduces results from our previous study [32], including the time-course of adaptation and de-adaptation (Figure 2) as well as the context-specific behavior associated with exposure to the dynamics of the object at a single orientation (Figure 3 and 4). In addition, adaptation and de-adaptation were found to occur at similar rates, which we show to be diagnostic of a single-rate process. Thus, in contrast to the dual-rate process thought to underlie adaptation to novel dynamics [17], adaptation to familiar dynamics appears to be mediated by a process which adapts at a single rate. We also confirm predictions of the model with two new experiments in which subjects were exposed to the dynamics of the object at multiple orientations (Figure 5 and 6). A key aspect of the model is that separate states are associated with different visual contexts of the object. This context-specific state represents the subject's estimate of object mass for a particular visual orientation. The state (mass estimate) is updated based on the kinematic error experienced on each trial and a generalization function determines how errors in the active context affect the states associated with the non-active contexts. In addition, each state undergoes spontaneous trial-based decay independent of the current context. Finally, by considering a version of the model in which the individual elements of the context-selection vector were free parameters, we show that the generalization function is Gaussian in form.

Having shown that the model can account for our previous results obtained during exposure to the dynamics of the object at a single orientation, we test predictions of the model with two new experiments in which subjects experienced the dynamics at multiple orientations. We first examined the case of dual-context exposure, in which the object alternated between two different orientations (0° and 180°). The model correctly predicted that, due to the relatively narrow generalization function, adaptation and de-adaptation would be partially independent in each context (Figure 5C and D, respectively). Specifically, after initial adaptation (or de-adaptation) in the first context, the model correctly predicted that there would be little benefit (transfer) to the second context. In addition, the spontaneous decay in the model predicted that small decreases in performance would occur each time the context alternated (Figure 5E). This partial de-adaptation in the non-active context was further examined in the second new experiment, in which the object switched randomly between five different orientations. In this case, the model correctly predicted that partial de-adaptation would occur in all non-active contexts, but would be greater for those contexts furthest removed from the active one (Figure 6). Taken together, the modeling and experimental results from the current study provide further support that internal models of familiar object dynamics are mediated my multiple context-specific representations.

Whereas a dual-rate process is thought to mediate adaptation to novel dynamics [17], results from the current study suggest that adaptation to the familiar dynamics of everyday objects is mediated by a single-rate process. Specifically, in the case of novel dynamics, a dual-rate model (DRM) has been proposed which has fast and slow adaptation processes acting in parallel [17]. Such a model explains various phenomena observed during adaptation to novel dynamic perturbations, such as fast de-adaptation, spontaneous recovery and savings (see Figure S3 and the associated discussion in Text S1). In Experiment 1 of the current study (Figure 2), we found no evidence for fast de-adaptation (see inset of Figure 2C). However, due to methodological constraints associated with our task, we were unable to test for spontaneous recovery and savings. Rather, in simulations and an extensive search of the DRM parameter space (see Figure S4 and Figure S5 of Text S1), we show that the absence of fast adaptation is a diagnostic feature of single-rate adaptation processes. Specifically, we show that DRM solutions which do not exhibit fast adaptation, exhibit neither spontaneous recovery nor savings. Such solutions are single-rate parameterizations of the DRM. Results of the current study thus suggest that the adaptation processes engaged when subjects are exposed to novel dynamics differ from the processes engaged during the manipulation of everyday objects.

Fast adaptation to the dynamics of familiar objects is consistent with the idea that there are two components to learning: structural learning and parameterization [41], [42]. In structural learning, the motor system extracts the structural form of the sensorimotor transformation that underlies a particular task. Once the structure has been learned, adaptation to different tasks that share the same structure can proceed rapidly through parameterization of the existing structure. When considering a set of similar objects, the structure can be represented by the form of the equations which describe the dynamics, whereas the parameters represent the values that vary across objects (such as mass and moment of inertia). As such, when manipulating familiar objects with dynamics which are captured by an existing structure, adaptation can be rapid because it primarily involves parameterization. For example, when lifting an object of unknown mass, subjects adapt their predictive load and grip forces rapidly within a few trials [5], [23], [24], [25]. Similarly, rapid adaptation of digit forces occurs when subjects lift objects with an asymmetrical centre of mass [26], [27], [28], [29]. Rapid adaptation was also observed in the current study, in which the time constant for adaptation was 3.3 trials. In contrast, when subjects are presented with objects which have novel and unusual dynamics they adapt much more slowly. For example, objects with internal degrees of freedom can take hundreds of trials to learn [43], [44]. Similarly, adaptation to novel state-dependent dynamics has a long time course [22]. For example, the time constant for adaptation to velocity-dependent curl fields is around 40 trials (see Figure S3-B of Text S1). We suggest that this longer time course reflects the requirement to learn the unfamiliar structure of the dynamics.

The formulation of the current model draws upon various state-space models that have been previously applied to adaptation in the motor system [15], [16], [17], [18], [19], [20]. For example, dynamic perturbation studies which examined velocity-dependent force-fields showed that context-specific adaptation could be modeled using a multiple-state model that included a generalization function tuned to the current context [15], [16]. In this case, the context was the target direction which thus involved a change in the kinematics of the movement. In contrast, in our experiments the movement kinematics were constant because the arm remained in the same configuration and subjects made the same movement on all trials. What varied in our experiments was the visual orientation of the object. Results from the current study show that, in addition to kinematic contexts and novel state-dependent dynamics, multiple-context state-space models can also be applied to visual contexts and the familiar dynamics of objects. The role of visual context was particularly striking in Experiment 2 (see Figure 3). In this experiment, subjects experienced the dynamics of the object at a single orientation only. The context-dependent behavior observed in this experiment was in response to changes in the visual orientation of the object; the dynamics and kinematics associated with probe trials remained constant. Moreover, based on fitting the model to this experiment, we were able to predict how subjects would behave when they experienced the dynamics of the object at multiple orientations.

Our results suggest that the dynamics of familiar objects are not represented globally, but rather as a set of local representations that are selectively engaged by the current context of the object (its visual orientation). Previous studies have also examined the ability of contextual cues to engage separate representations in the motor system. For example, a number of studies have focused on the ability of subjects to learn opposing dynamic or kinematic perturbations, in which the direction of the perturbation alternates over successive blocks. In the absence of an appropriate contextual cue, the opposing perturbations compete for a single representation and concurrent adaptation is not possible [45], [46], [47], [48], [49], [50], [51], [52]. However, if a suitable contextual cue is associated with each perturbation, separate representations are engaged and concurrent adaptation can be achieved [19], [20], [53], [54], [55], [56], [57]. In the current study, the dual-context experiment similarly required subjects to adapt to opposing dynamic perturbations because the forces produced by the object reversed direction as it alternated between 180° and 0° (Figure 5). In this case, however, subjects had perfect contextual information (the visual orientation of the object) which allowed them to produce forces in the appropriate direction from the very first trail. Rather, the observed changes in performance when the context switched (Figure 5E) were associated with adaptation (and de-adaptation) of force magnitude.

Of relevance to the current study, two previous studies of object manipulation have also characterized the rate of adaptation and de-adaptation [30], and the pattern of generalization [31] of familiar object dynamics. In these previous studies, grip force adaptation was examined during a bimanual object manipulation task. Importantly, the current model successfully reproduced key features from these previous results (Figure S6 in Text S1). Specifically, results from four experiments (two experiments from each previous study) were concurrently fit by the model, which reproduced the time-course of adaptation and de-adaptation reported in the first study and the local context-dependent pattern of generalization reported in the second study. Interestingly, the rate of de-adaptation of grip force in the first study was found to be slower than the rate of adaptation [30]. This result could be reproduced by the current model by assuming that grip force de-adaptation is not an active error-driven process, but rather occurs through passive decay (as captured by the retention constant). This assumption seemed justified because, in the task examined by these previous studies, producing a grip force response on the unlinked de-adaptation trials did not cause the virtual object to displace. In the absence of a kinematic error, de-adaptation in the task may thus rely on passive trial-by-trial decay. The relatively low value for the retention constant (*α* = 0.8122) obtained when fitting the model is consistent with this suggestion, which would allow grip force to rapidly decay. Similarly, the relatively high learning-rate constant (*β* = 0.2568) would allow grip force to adapt quickly despite this high rate of passive decay. In the current study, passive decay was also found to be an important process, responsible for the progressive de-adaptation of non-active contexts despite ongoing exposure to the object.

We have suggested that because the structure of dynamics which are familiar is already represented by the sensorimotor system, adaptation is rapid and engages a single-rate process. This is in contrast to adaptation to novel force-fields, which proceeds more slowly and is mediated by a dual-rate process. However, this view is not the only interpretation of our results. For example, adaptation to the dynamics of the object in our task may be inherently easier. Alternatively, there may be something inherently difficult about adapting to velocity-dependent force-fields. This argument is weakened by the variety of previous studies reviewed above. Specifically, in studies of familiar dynamics, adaptation is always rapid. Moreover, for those cases of familiar dynamics examined in the current study (the current task, and the bimanual object manipulation and lifting tasks in Figure S6 and Figure S7 in Text S1, respectively) such adaptation appears to be mediated by a single-rate process. In contrast, in previous studies of novel dynamics reviewed above, adaptation is always slow, and in the case of velocity-dependent force-fields, is mediated by a dual-rate process. In further support of our view, the rate of adaptation to a sensorimotor task can increase dramatically when subjects become familiar with the structure of the task [41]. Thus, we hypothesize that familiarity plays a key role in determining the rate of adaptation and may explain the observed differences in the processes which mediate adaptation to familiar versus novel dynamics.

In summary, by using state-space models, the current study has highlighted similarities and differences in the processes which mediate adaptation to novel and familiar dynamics. In both cases, adaptation is mediated by multiple context-specific representations. In the case of novel dynamics, these representations are selected based on the kinematic context of the movement. In the case of familiar object dynamics, the representations can be selected based on the visual context of the object. And whereas the relatively slow adaptation to novel dynamics is mediated by a dual-rate process, the rapid adaptation observed when subjects manipulate objects with familiar dynamics appears to be mediated by a single-rate process. Thus, the human ability to skillfully manipulate objects appears to be mediated by multiple representations. These representations, which capture the local dynamics associated with specific contexts of the object, can be selectively engaged by visual information alone, and are updated based on the dynamics of specific objects via a single-rate adaptation process.

## Supporting Information

Text S1 Supporting information and additional analyses, including seven figures (Figure S1 to Figure S7).
(DOC)
