# Supplementary material for: A Single-Rate Context-Dependent Learning Process Underlies Rapid Adaptation to Familiar Object Dynamics
Source: PLoS Comput Biol. 2011 Sep 29;7(9):e1002196. doi: 10.1371/journal.pcbi.1002196 (PMC3182866; doi:10.1371/journal.pcbi.1002196)
Supplement: Text S1 — Supporting information and additional analyses, including seven figures (Figure S1 to Figure S7). (DOC) [file pcbi.1002196.s001.doc]

**Supporting Information**

**A single-rate context-dependent learning process underlies rapid adaptation to familiar object dynamics**

James N Ingram, Ian H Howard, J Randall Flanagan and Daniel M Wolpert

**Supplemental Methods**

Figure S1 shows an analysis of the kinematics as well as the forces and torques generated by the WristBOT during the task for a typical trial. This verified that the WristBOT was able to accurately simulate the dynamics of the object.

**Experiment S1 – Estimating the compliance function**

The direction of the forces associated with rotating the object varies with object orientation (Figure 1B and Equation 4 in the main text). Thus, when experiencing the object at different orientations, the subject’s arm will be exposed to forces which perturb it in different directions. Given the variable compliance of the arm associated with perturbations in different directions (Franklin and Milner 2003; Gomi and Osu 1998), when all other factors remain constant, the peak displacement measured during the experiment may vary with object orientation simply as a result of the variable compliance of the arm. In order to extend the SRM to examine multiple object orientations, it was therefore necessary to estimate the compliance associated with different orientations of the object. Specifically, we define a compliance-dependent error function for the model as follows:

(S1)

The function defines the peak displacement or error (*e*) on trial *n* for a given orientation of the object (*θ*). As in the original error function (Equation 6 in the main text), the error is due to the difference between the actual mass of the object ( *f*) and the subject’s estimate of the mass (*x*). In this case, however, the error is the product of this difference and the compliance factor (*k*). The subscripts and superscripts on *k* in Equation S1 allow the compliance to vary for different orientations of the object (*θ*) and for positive (*f* > *x*) and negative (*f* < *x*) errors, respectively. This latter feature of the function allows the compliance to be different during adaptation and de-adaptation. Specifically, during adaptation, displacement of the handle is due to the object producing net forces on the subject. This is the case where the actual mass of the object ( *f*) is greater than the subject’s estimate of the mass (*x*). In contrast, during de-adaptation, displacement of the handle is due to the subject producing net forces on the object. This is the case where the actual mass is less that the subject’s estimate of the mass.

In order to estimate the compliance factor (*k*), a modified version of the basic SRM (Equation 5 in the main text) was fit to experimental data. In this modified model, the basic error function (Equation 6 in the main text) was replaced by the compliance-dependent error function (Equation S1). Subjects (n=12) experienced the object at 5 different orientations (0, -45, -90, -135 and 180°). They adapted and then de-adapted to the dynamics of the object 3 times at each orientation (Figure S2-A). Specifically,


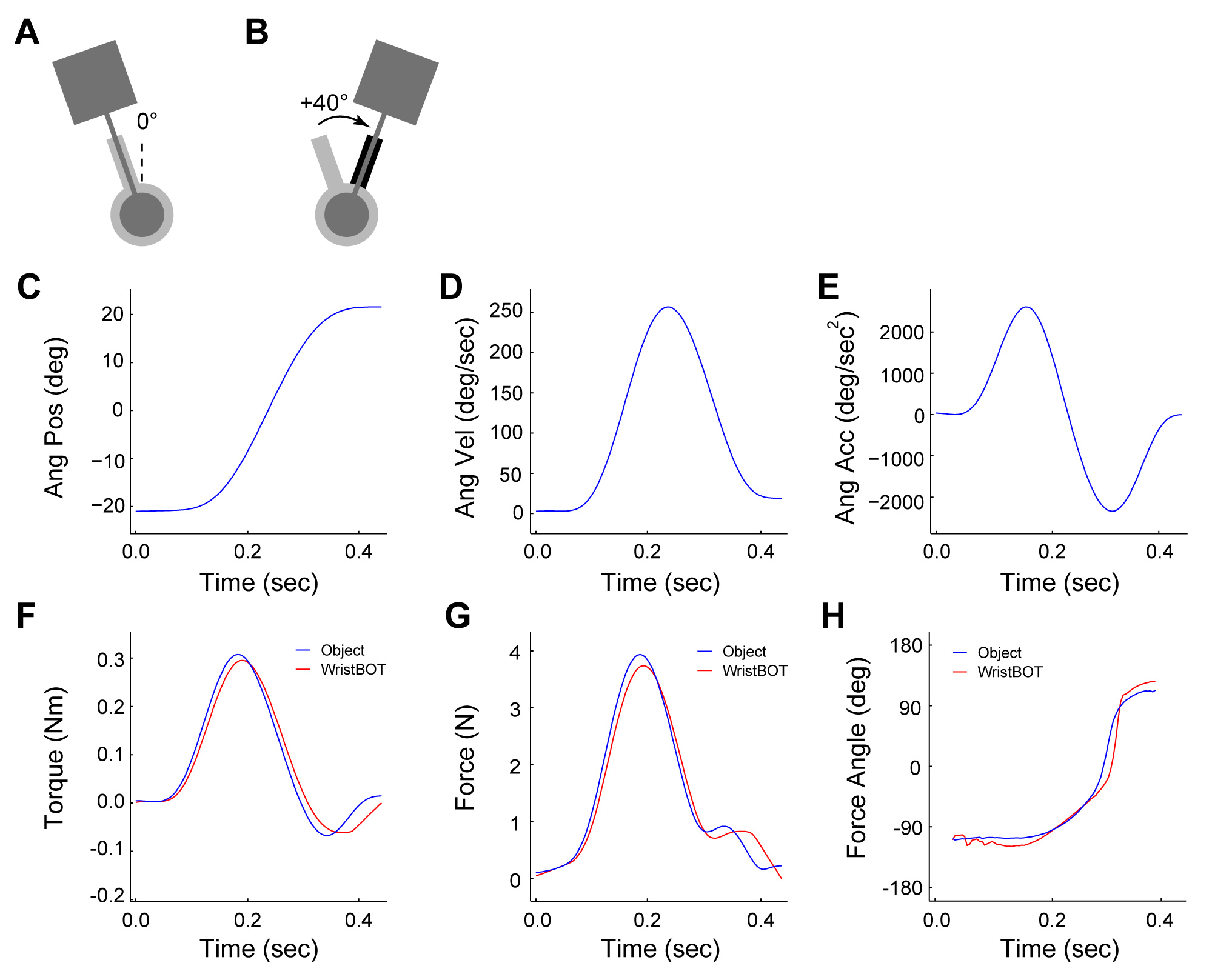


**Figure S1** Supplemental methods. **A** A CW rotation trial at 0° began with the object (dark grey) positioned in the home position (light grey disc) and aligned with the -20° target (light grey bar). **B** Movement onset was cued by a tone and the appearance of the +20° target (black bar). The subject then rotated the object +40° to reach the target. **C** The angular position of the object for a CW trial recorded from the WristBOT during an experiment with a typical subject. **D** The angular velocity of the object plotted as in panel C. **E** The angular acceleration of the object plotted as in panel C. **F** The torque associated with a CW trial. The red trace shows the torque simulated by the WristBOT. The blue trace shows the torque predicted from the kinematics shown in panels C, D and E. **G** The force magnitude associated with a CW trial plotted as in panel F. **H** The force direction associated with a CW trial plotted as in panel F.

in cycles of 138 trials for each orientation, subjects first completed 18 pre-exposure trials (zero-force), followed by 3 repeat blocks of 40 trials which consisted of 22 adaptation trials (full dynamics with 2 error-clamp trials at trial 16 and 17) and 18 de-adaptation trials (zero-force). Subjects thus completed a total of 690 trials (5 orientations and 18 + 3 x 40 = 138 trials per orientation = 690 trials). The presentation of object orientation was pseudo-randomized for each subject.

The peak displacement trial series for the 3 adaptation / de-adaptation blocks for each orientation were averaged for each subject and then across subjects. Composite blocks of 46 trials per orientation were then constructed from the final 8 pre-exposure trials, the 20 adaptation trials (the 2 error-clamp trials were omitted) and the 18 de-adaptation trials (8 + 20 + 18 = 46 trials). The mean peak displacement for the final 8 pre-exposure trials was subtracted from each block. The peak displacement data across the blocks of 46 trials for each orientation are shown in Figure S2-B (black trace). The expected pattern of adaptation and subsequent de-adaptation can be seen in the figure for each orientation. Also of note is the fact that peak displacement varied substantially with object orientation, as was expected due the variable compliance of the arm and the biomechanics of the task.

The modified SRM model which included the compliance-dependent error function was then fit to the experimental data in Figure S2-B. The model fit the *α* and *β* parameters from the SRM along with 10 parameters which implemented the compliance function. These 10 parameters were associated with the 5 orientations of the object, with separate values for positive errors (adaptation; *k^+^* in Equation S1) and negative errors (de-adaptation; *k^-^* in Equation S1), as explained above (5 orientations x 2 values per orientation = 10 parameters).

Overall, the model well captured the experimental data (Figure S2-B red trace, R^2^=0.8622). There was a small departure of the model from the experimental data during late adaptation for 180°, but otherwise the SRM combined with the compliance-dependent error function closely matched the peak displacement trial series during both adaptation and de-adaptation across the range of object orientations tested. The best-fit values for the SRM parameters were *α*=0.9733 and *β*=0.1874. The best-fit values for the compliance factor are shown in Figure S2-C (adaptation *k^+^*) and S2-D (de-adaptation *k^-^*).


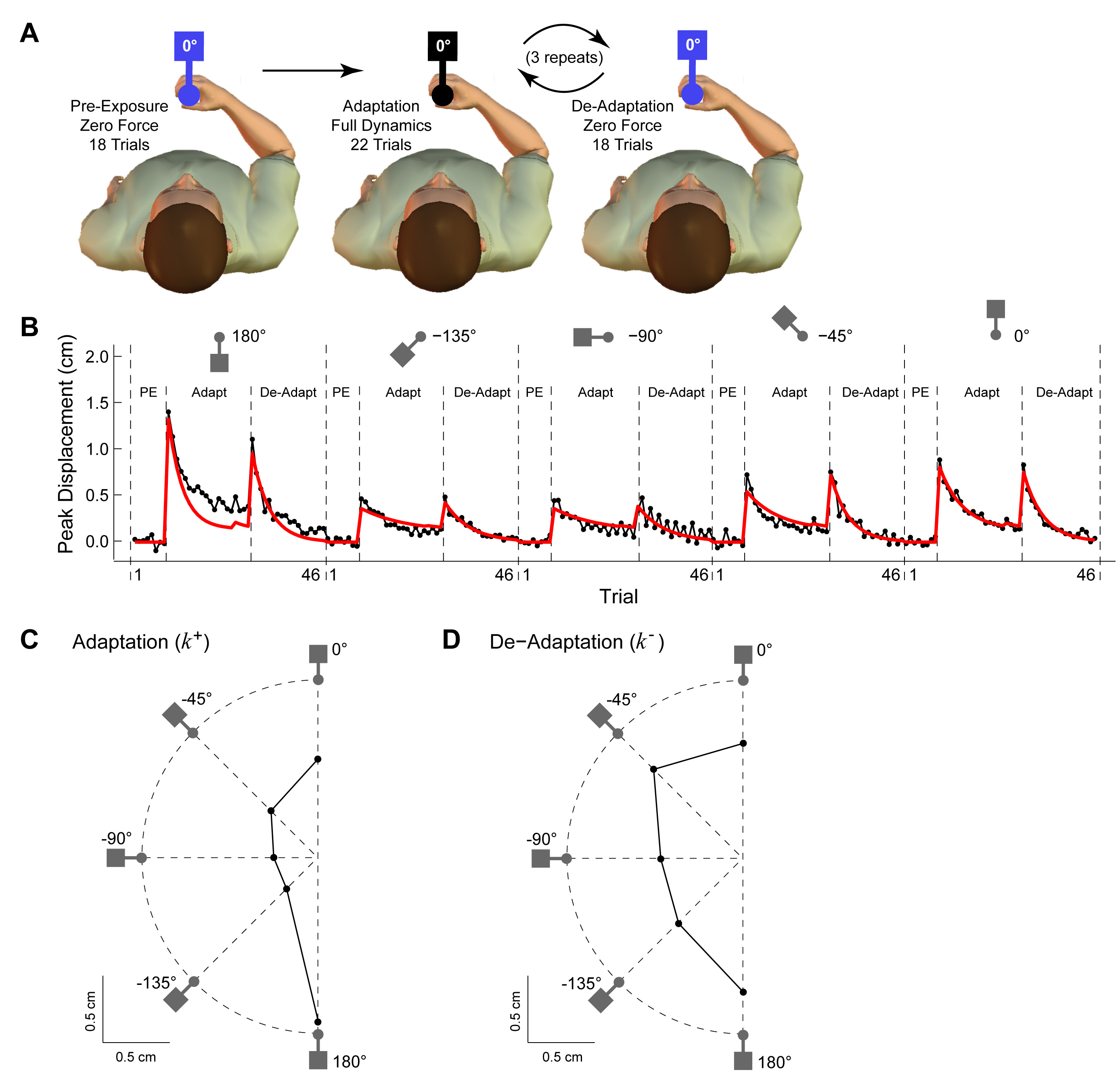


**Figure S2** Estimating the compliance function. **A** Subjects (n=12) completed cycles of 136 trials at each of 5 different orientations (0, -45, -90, -135, 180°). The trial sequence is shown for 0° and consists of a pre-exposure of 18 trials followed by 3 repeated blocks of 22 adaptation and 18 de-adaptation trials (18 + 3 x 40 = 136). **B** The peak displacement for composite blocks of 46 trials for each orientation (PE = Pre-Exposure). Composite blocks consist of the final 8 pre-exposure trials followed by the mean of the 3 repeat blocks of adaptation / de-adaptation (the 2 error-clamp trials during adaptation have been omitted). The black trace shows the peak displacement trial series from the experiment averaged across subjects. The red trace shows the model fit. **C** The best-fit values for the adaptation compliance factor (*k^+^* in Equation S1) for different orientations of the object. The units are peak displacement (cm) per unit of adaptation error in the model (which is dimensionless). **D** The best-fit values for the de-adaptation compliance factor (*k^-^* in Equation S1) for different orientations of the object, plotted as in panel C.

**Single-rate versus dual-rate adaptation processes**

When the SRM and DRM were fit to the data from Experiment 1 (Figure S3-A; see also Figure 2 in the main text), the two models provided almost identical fits (SRM R^2^=0.7131, DRM R^2^=0.7153). However, the BIC analysis selected the 2-parameter SRM because the slightly better fit provided by the 4-parameter DRM did not justify its additional parameters. Based on this result, we might conclude that adaptation to the dynamics of familiar objects is mediated by a single-rate process. This would be contrary to results which suggest that adaptation to novel dynamics is mediated by a dual-rate process (Smith et al. 2006). Before making this conclusion, however, it is necessary to show that the paradigm implemented in Experiment 1 can distinguish between single-rate and dual-rate adaptation processes.

In previous studies, phenomena such as fast de-adaptation, spontaneous recovery and savings have been associated with dual-rate adaptation processes (Smith et al. 2006). In the following sections, we examine these phenomena along with other evidence from Experiment 1 which supports our conclusion that a single-rate process mediates adaptation to familiar dynamics.

**Fast de-adaptation**

When subjects adapt and subsequently de-adapt to novel dynamics (such as a velocity-dependent curl-field), the rate of de-adaptation is faster than the rate of initial adaptation (Davidson and Wolpert 2004; Shadmehr et al. 1998; Smith et al. 2006). This phenomenon cannot be reproduced by the SRM and has been argued to be a feature associated with dual-rate adaptation processes (Smith et al. 2006). Importantly, fast de-adaptation was not observed in Experiment 1 in the current study, which exhibited similar rates of adaptation and subsequent de-adaptation (see inset of Figure S3-A). Specifically, we fit exponential functions (see Equation 16 in the main text) individually to each subject’s data to determine the time constants of adaptation and de-adaptation. The mean time constants for adaptation (3.3±1.1 trials) and de-adaptation (4.0±1.1 trials) did not differ significantly (paired two-tailed t-test, p=0.21).

The phenomenon of fast de-adaptation was further examined by simulating an adaptation / de-adaptation paradigm using the DRM with the best-fit parameters from Experiment 1 (*α_1_*=0.9808, *β_1_*=0.0139, *α_2_*=0.9453, *β_2_*=0.2053). We also simulated a DRM with the parameters obtained in a previous study (Smith et al. 2006) which examined adaptation to novel dynamics implemented by a velocity-dependent curl-field (*α_1_*=0.992, *β_1_*=0.020, *α_2_*=0.590, *β_2_*=0.210). This allowed us to directly compare the behavior of the DRM for parameters obtained for familiar and novel dynamics (Figure S3-B). Fast de-adaptation can be quantified by determining the ratio of the exponential time constants of adaptation and de-adaptation (A/DA). Values close to 1 indicate similar rates of adaptation and de-adaptation, whereas values greater than 1 indicate faster de-adaptation. For the DRM simulated with parameters from Experiment 1 in the current study of familiar dynamics, this ratio is 1.0 (blue trace in Figure S3-B). For the DRM simulated with parameters from the previous study of novel dynamics, this ratio is 1.5 (red trace in Figure S3-B).

**Spontaneous recovery and savings**

Spontaneous recovery and savings have also been argued to be key features of a dual-rate adaptation process (Smith et al. 2006). Unfortunately, it is difficult to implement the paradigms that would test for these phenomena in our object


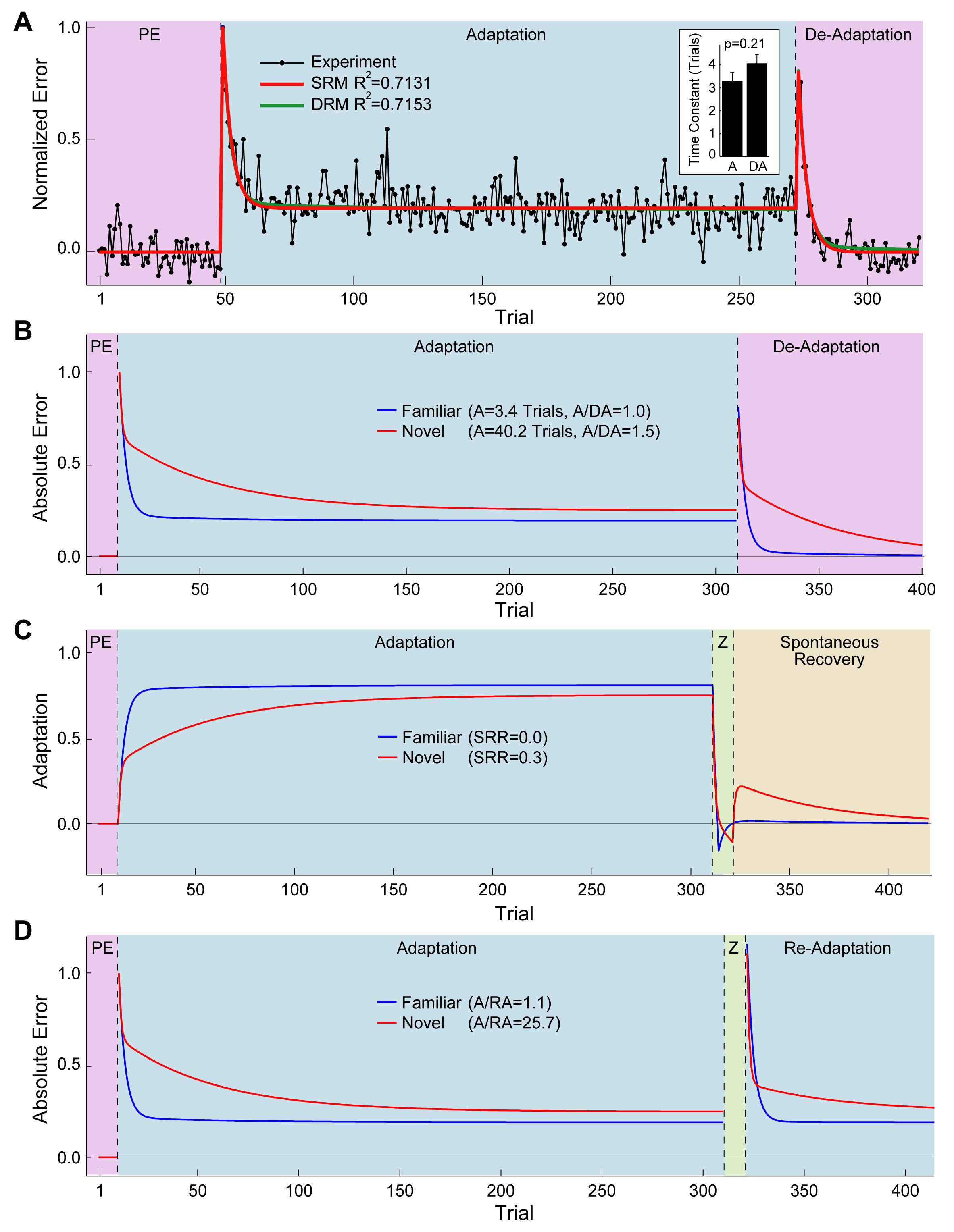


**Figure S3** Phenomena associated with dual-rate adaptation processes. **A** SRM (red trace) and DRM (green trace) fits to normalized peak displacement from Experiment 1 from the current study (black trace). Inset shows the mean exponential time constant (and standard error; n=8) for adaptation (A) and de-adaptation (DA). PE = Pre-exposure. **B** DRM simulations of an adaptation / de-adaptation paradigm. The two traces show simulations using parameters obtained from fitting the DRM to Experiment 1 from the current study of familiar object dynamics (blue trace) and parameters obtained from a previous study of novel dynamics (red trace). The values shown in the key are the rates (exponential time-constants) of adaptation (A) and the ratio of the rates of adaptation and de-adaptation (A/AD). A/AD values close to 1 indicate little or no fast de-adaptation. **C** DRM simulations of a spontaneous recovery paradigm, plotted as in panel B. The values shown in the key are the ratios of the peak adaptation during spontaneous recovery relative to the final level of adaptation (SRR). SRR values close to 0 indicate little or no spontaneous recovery. **D** DRM simulations of a savings paradigm, plotted as in panel B. The values shown in the key are the ratios of the rate of initial adaptation relative to the rate of re-adaptation (A/RA). A/RA values close to 1 indicate little or no savings.

manipulation task. However, the appropriate experiments can be simulated using the parameters obtained from fitting the DRM to Experiment 1, as described above.

Spontaneous recovery can be quantified by the ratio of the peak adaptation during the recovery phase relative to the final adaptation in the adaptation phase (SRR, see Figure S3-C). In this case, values close to zero indicate no spontaneous recovery, whereas values greater than zero indicate greater degrees of spontaneous recovery. Similarly, savings can be quantified by the ratio of the rate of initial adaptation relative to the rate of re-adaptation (A/RA, see Figure S3-D). In this case, values close to 1 indicate no savings, whereas values greater than 1 indicate greater degrees of savings. Both spontaneous recovery (SRR=0.3) and savings (A/RA=25.7) occur in DRM simulations which use the parameters previously obtained for novel dynamics (red traces in Figure S3-C and D, respectively). However, when DRM simulations are performed with the parameters obtained from Experiment 1 in the current study, neither spontaneous recover (SRR=0.0) nor savings (A/RA=1.1) are observed (blue traces in Figure S3-C and D, respectively). This result would be expected if the DRM is fit to data generated by a singe-rate adaptation process.

**Parameter redundancy**

We performed an extensive analysis of the parameter space of the SRM and DRM (Figure S4). In the case of the 2-dimensional parameter space of the SRM, we find a narrow range of parameters which yield an R^2^ within 1% of the best-fit value for Experiment 1 (bottom left inset of Figure S4). In contrast, for the 4-dimensional parameter space of the DRM, we find a wide range of solutions spanning the parameter space which yield an R^2^ within 1% of the best-fit value for Experiment 1. Specifically, in DRM simulations in which we test over 24x10^6^ distinct points across a wide range of parameter space, we find over 19,000 solutions within 1% of the best-fit R^2^. This suggests that when fitting data from Experiment 1, the 4 parameters of the DRM are somewhat redundant, as would be expected if a DRM is fit to data generated by a single-rate process. Importantly, none of the 1% best-fit DRM solutions exhibit fast de-adaptation, spontaneous recovery or savings when the appropriate experiments are simulated. This suggests that the results of Experiment 1 constrain DRM solutions to regions of the parameter space which do not exhibit phenomena normally associated with dual-rate adaptation processes. We explore this point further in the following section.

**The absence of fast de-adaptation is diagnostic of a single-rate process**

Finally, we examined the relationship between the various phenomena which are associated with dual-rate adaptation processes. Specifically, we analyzed results of the parameter search described above in order to determine how fast de-adaptation relates to both spontaneous recovery and savings. If the absence of fast de-adaptation (as was shown in Experiment 1) is diagnostic of a single-rate process, there should be a relationship between the measure which quantifies fast de-adaptation (A/DA) and the measures which quantify spontaneous recovery (SRR) and savings (A/RA). This relationship can be seen in Figure S5. The figure shows that DRM solutions which do not exhibit fast de-adaptation (A/DA values close to 1) exhibit neither spontaneous recovery (SRR values close to 0; Figure S5-A) nor savings (A/RA values close to 1; Figure S5-B). Specifically, the colored (non-black) regions of these plots show that increasing values of A/DA are associated with increasing values of both SRR and A/RA. Moreover, the DRM solutions which yield the best fits to Experiment 1 (within the top 1% of R^2^ values, as described above) are all located in the single-rate region of the plots which exclude fast de-adaptation, spontaneous recovery and savings (see the ­­­­­“warm” colored regions in Figure S5-A and B). Thus, although the DRM solutions which yield a good fit to the data from Experiment 1 span a wide range of the DRM parameter space (Figure S4), when plotted using co-ordinates which characterize dual-rate phenomena, these solutions cluster in the single-rate region of the space (Figure S5-A and B). In contrast, the DRM solutions which yield the top 1% of fits to simulated velocity-dependent curl-field data (Smith et al. 2006) are all located in dual-rate regions of the space in which the three phenomena co-occur (see “warm” colored regions in Figure S5-C and D).

Overall, the arguments and analyses presented here and in the main text provide good support for excluding a dual-rate adaptation process based on results from Experiment 1. This suggests that the adaptation to familiar object dynamics is mediated by a single-rate process.


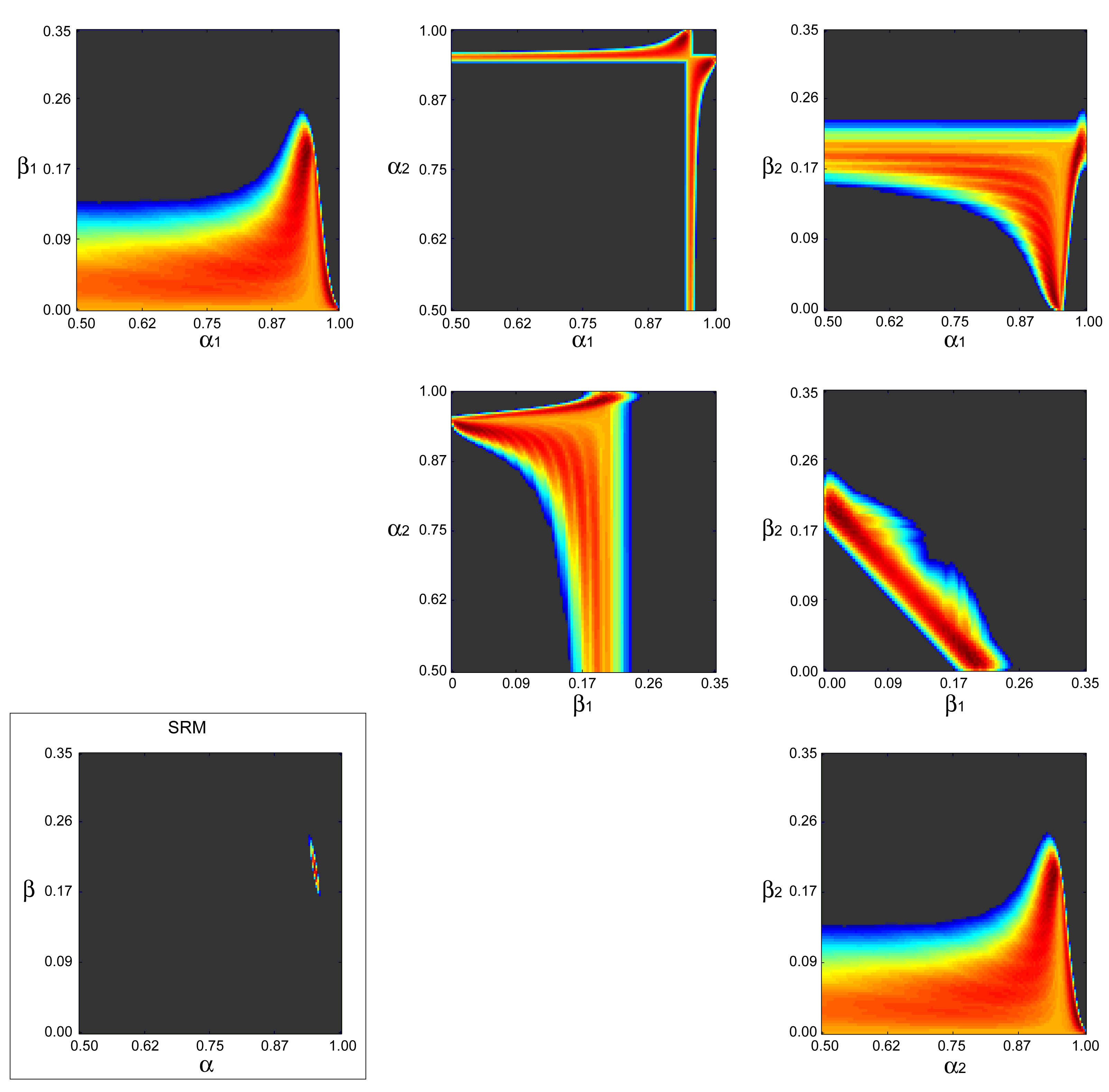


**Figure S4** The SRM and DRM parameter space analysis. The colored regions in each panel show the model parameters which yield an R^2^ value within 1% of the best-fit solution for Experiment 1. The inset in the bottom left shows the 2-dimensional (2D) parameter space of the SRM. The other panels show 2D views of the 4-dimensional parameter space of the DRM.


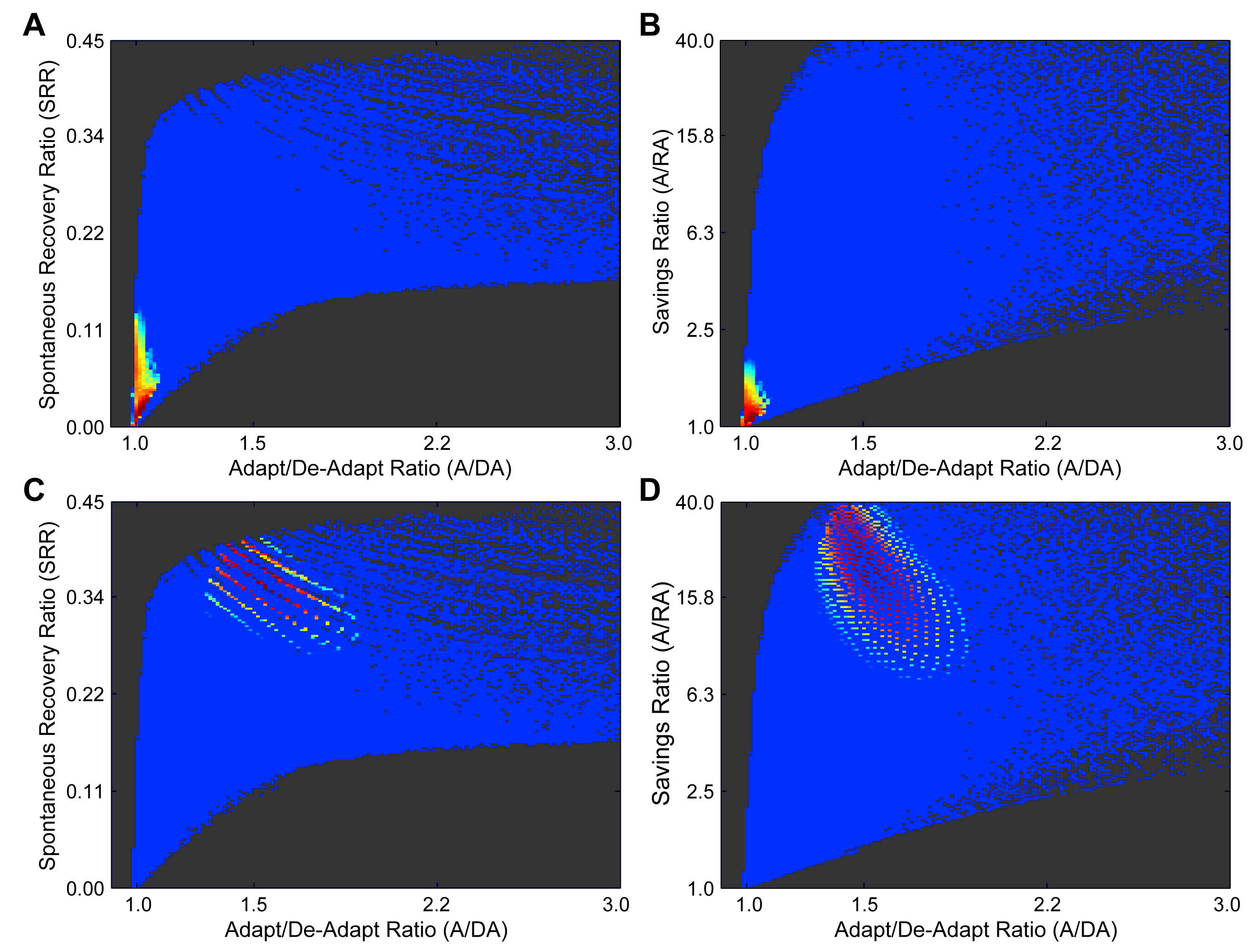


**Figure S5** The relationship between the various DRM phenomena. **A** The relationship between the adaptation / de-adaptation ratio (A/AD) and the spontaneous recovery ratio (SRR). No DRM parameters yielded points in the black region. The warm colors indicate the top 1% of R^2^ values for DRM fits to Experiment 1. **B** The relationship between the adaptation / de-adaptation ratio (A/AD) and the savings ratio (A/RA), plotted as in panel A. **C** The relationship between the adaptation / de-adaptation ratio (A/AD) and the spontaneous recovery ratio (SRR). The warm colors indicate the top 1% of R^2^ values for DRM fits to simulated velocity-dependent curl-field data (see text for details). **D** The relationship between the adaptation / de-adaptation ratio (A/AD) and the savings ratio (A/RA), plotted as in panel C.

**Previous studies of object manipulation**

A number of previous studies of object manipulation are of particular relevance to the current study. For example, Witney and colleagues examined grip-force modulation during bimanual object manipulation and characterized the time-course of adaptation and de-adaptation (Witney et al. 2000) as well as the context-specific pattern of generalization (Witney and Wolpert 2003). These previous studies showed that grip force develops and decays progressively over a number of trials, and that the pattern of generalization is dependent on the contexts in which the object is experienced (in this case, the movement direction). They thus provide an opportunity to test the current model using a dataset obtained from a distinct object manipulation task.

Previous studies in which subjects lift an object with an asymmetrically offset centre of mass (Bursztyn and Flanagan 2008; Fu et al. 2010; Salimi et al. 2000; Zhang et al. 2010) are also relevant because the dynamics of the lifting task share important features with the object rotation task from the current study (see below). In addition, the time-course of adaptation during asymmetric object lifting has recently been characterized (Fu et al. 2010).

**Bimanual object manipulation**

The essential features of the bimanual object manipulation task are shown in Figure S6-A (for full details see Witney et al. 2000; Witney and Wolpert 2003). Briefly, two force actuators simulated the linkage dynamics of a virtual rigid object that was grasped at each end by the left and right hands using a precision grip. On linked trials, the force produced by the active left hand when subjects pulled on the simulated object was transmitted to the passive right hand (consistent with grasping a rigid object between the two hands). On unlinked trials, the active force was not transmitted. Subjects were instructed to pull on the object with the left hand and stabilize it with the right hand. The grip-force response of the passive right hand was measured by a force sensor (see Figure S6-A). On unlinked trials, this grip-force response represents a predictive response by subjects. In this respect, it is similar to the forces measured on error-clamp trials in the current study.

In experiments characterizing the adaptation and de-adaptation of the grip-force response (Witney et al. 2000), subjects experienced either 1 linked trial (Figure S6‑B) or 3 linked trials (Figure S6-C) followed by 6 unlinked trials. In experiments characterizing the context-specific pattern of generalization (Witney and Wolpert 2003), subjects experienced 60 linked trials, generating active force either in 1 direction (Figure S6-D) or 2 directions (Figure S6-E). The pattern of generalization was then characterised using unlinked probe trials in 8 directions. Linked trials in the training directions were included with the probe trials to prevent de-adaptation. The MCSRM4 was concurrently fit to results from these four experiments as described below.

Peak grip force represents the output of subjects in the four experiments (see Figure S6-B to E) and so the adaptation state of the model was fit to the experimental grip force data. This is in contrast to the experiments described in the main text, where the model error was fit to peak displacement data. However, despite this difference, model fitting proceeded as described in the main text. Grip force data across the two studies was digitized from the original figures using screen based digitization software in which each point on the enlarged figure was entered manually using the mouse. These values for peak grip force were normalized to a maximum value of 7 N across all experiments. In addition, the maximum grip force between the two experiments in each study was normalized in order to account for small subject-to-subject variability. This resulted in a normalized grip-force vector which consisted of data from the four experiments. The adaptation state of the MCSRM4 was then concurrently fit to the normalized grip force vector. In this case, the MCSRM4 used the basic error function (Equation 6) and the direction of movement of the active hand provided the object context.

**Results**

The model successfully fit the results across all experiments (*R^2^*=0.8419). Specifically, the model reproduced the time-course of adaptation and de-adaptation in the 1-Link and 3-Link experiments (Figure S6-B and C, Witney et al. 2000). The model also reproduced the context-specific pattern of generalization in both 1-Direction and 2-Direction experiments (Figure S6-D and E, Witney and Wolpert 2003). The best-fit parameters were *α*=0.8122, *β*=0.2568, *σ*=18.2° and *d*=0.16. A comparison of the parameters associated with the generalization functions for bimanual grip force adaptation and the object rotation task from the current study can be seen in the inset of Figure S6-E.

In the 1-Link / 3-Link experiments of the original study, the time-course of de-adaptation was found to be slower than the initial adaptation. This was reproduced by the model by assuming that de-adaptation on unlinked trials occurs via passive trial-by-trial decay, rather than an active error-driven processes. This is consistent with the idea that unlinked trials are equivalent to error-clamp trials in the current study, as discussed above. To specifically characterize the time-course of adaptation and de-adaptation, an exponential function was fit (as described in the main text) to the output state of the model in the 3-Link experiment (see Inset of Figure S6-C). The adaptation of grip force in this case was found to have an exponential time-constant of 0.9 trials, whereas the time-constant for de-adaptation was 4.8 trials.

**Asymmetric object lifting**

A task in which subjects lift an object with an asymmetrically offset centre of mass (Figure S7-A) has been examined in various previous studies (Bursztyn and Flanagan 2008; Fu et al. 2010; Salimi et al. 2000; Zhang et al. 2010). The task involves minimizing rotational displacement (tilt) when lifting the object and requires subjects to generate a compensatory torque to oppose the torque associated with the offset centre of mass (see inset of Figure S7-A). The dynamics of the task can be considered to be the translational equivalent of the rotation task from the current study. In both tasks, subjects predictively generate either a torque (during lifting) or a force (during rotation) to stabilize the object against the destabilizing effects of its first moment (for full details see Figure S7-B).

The time-course of compensatory-torque adaptation when lifting an asymmetrical object has been recently characterized (Fu et al. 2010). To facilitate comparison across the different object manipulation tasks considered in the current study, exponential functions were fit (as described in the main text) to data from this previous study (Figure S7-C and D). The exponential time-constant for compensatory torque adaptation in two different object conditions (“CM-Left” in Figure S7-C; “CM-Right” in Figure S7-D) was 0.9 trials.


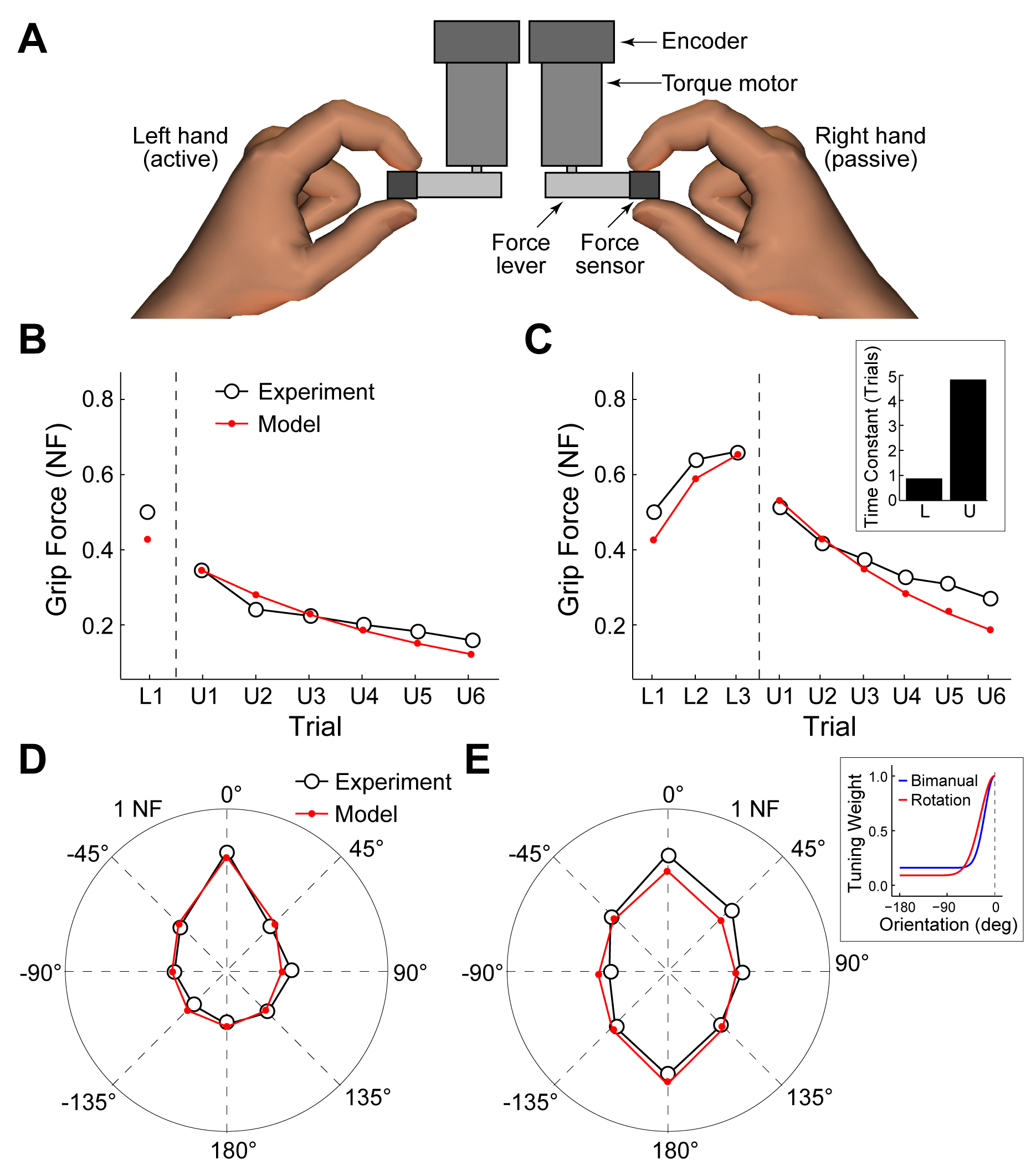


**Figure S6** Grip force adaptation during bimanual object manipulation (Witney et al. 2000; Witney and Wolpert 2003). **A** Subjects grasped the left and right force actuators with each hand using a precision grip. The actuators simulated a virtual object grasped bimanually. A 6-axis force / torque sensor measured grip force for each hand. The left (active) hand generated forces on the object. The right (passive) hand was required to stabilize the object. On linked trials (L), left-hand forces were transmitted to the right hand. This resulted in a progressive adaptation of right-hand grip-force responses. On unlinked trials (U), left-hand forces were not transmitted. This resulted in a progressive de-adaptation of right-hand grip force responses. **B** The 1‑Link adaptation experiment (Witney et al. 2000). Subjects experienced 1 linked trial, followed by 6 unlinked trials. Experimental grip force data (black symbols) are normalized values re-plotted from the original figure. Model data (red symbols) was obtained by concurrently fitting the MCSRM4 to the adaptation / de-adaptation experiments (Witney et al. 2000) and the generalization experiments (Witney and Wolpert 2003). **C** The 3-Link adaptation experiment, plotted as in panel B. Subjects experienced 3 linked trials, followed by 6 unlinked trials. Inset shows exponential time-constant of adaptation on linked trials (L) and subsequent de-adaptation on unlinked trials (U). **D** The 1‑Direction generalization experiment from (Witney and Wolpert 2003). Subjects generated active force in a single direction (0°) for 60 linked trials. Generalization was then probed using unlinked trials in 8 directions. Experimental grip force data (black symbols) are normalized values re-plotted from the original figure. Model data (red symbols) was obtained by concurrently fitting the MCSRM4 to all experiments, as described in panel B. Outer circle is 1 normalized force unit (NF). **E** The 2-Direction generalization experiment, plotted as in panel D. Subjects generated active force in 2 directions (0° and 180°) for 60 linked trials. Generalization was then probed as described in panel D. The inset compares the MCSRM4 generalization functions for the bimanual object manipulation task (Witney et al. 2000; Witney and Wolpert 2003) and the object rotation task (from the current study).


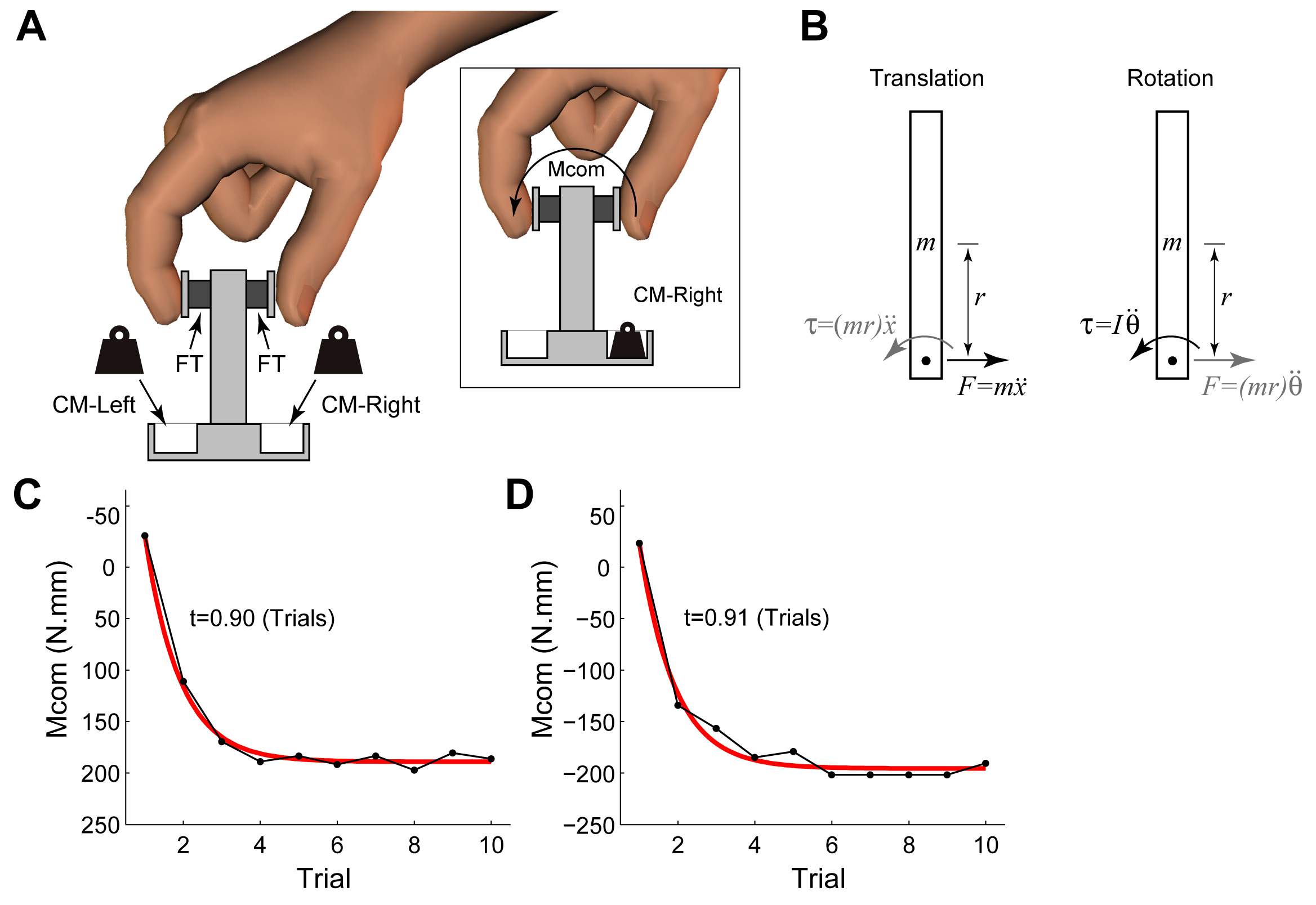


**Figure S7** Lifting objects with an asymmetrical centre of mass (Fu et al. 2010; Zhang et al. 2010). **A** Subjects lifted an invert T-shaped object using a precision grip. The thumb and index finger contacted the object via high-friction grip surfaces attached to separate 6-axis force / torque sensors (FT). The centre of mass (CM) of the object could be located asymmetrically to the left or right by placing a weight in the receptacles as shown. There were no visual cues of the CM location. The task was to lift the object vertically while minimizing the tilt caused by the asymmetric placement of the CM. Inset shows CM‑Right condition which required subject to generate a counter-clockwise compensatory torque (M_com_) to prevent clockwise tilt of the object. **B** Comparison of the dynamics of object translation (lifting, as in Fu et al. 2010) and object rotation (as in the current study). Gravity has been omitted for simplicity. Large black arrows and black equations show the force ( *F* ) and torque (τ) required to translate ($\ddot{x}$) and rotate ($\ddot{\theta}$) the object according to its inertia (*m*) and moment of inertia (*I* ), respectively. If the translating force or rotating torque is applied at the centre of mass of the object (*m*), the moment arm (*r*) will have zero length and the first moment (*mr*) will be zero. Otherwise, for a non-zero first moment, the object will generate a torque when translated and a force when rotated, as shown by the grey arrows and grey equations. Studies show that, in both cases, subjects predictively generate torques and forces to stabilize the object against the destabilizing influence of the first moment. **C** Compensatory torque (M_com_) generated by subjects who lifted the CM-Left object (clockwise positive). Torque was measured by the force / torque sensors at the time of object lift-off. Black points show mean subject data (re-plotted from Fu et al. 2010). Red line shows exponential fit (t = time constant). **D** Compensatory torque (M_com_) generated by subjects who lifted the CM-Right object (counter-clockwise negative), plotted as in panel C.

References

**Bursztyn LL, and Flanagan JR**. Sensorimotor memory of weight asymmetry in object manipulation. *Exp Brain Res* 184: 127-133, 2008.

**Davidson PR, and Wolpert DM**. Scaling down motor memories: de-adaptation after motor learning. *Neurosci Lett* 370: 102-107, 2004.

**Franklin DW, and Milner TE**. Adaptive control of stiffness to stabilize hand position with large loads. *Exp Brain Res* 152: 211-220, 2003.

**Fu Q, Zhang W, and Santello M**. Anticipatory planning and control of grasp positions and forces for dexterous two-digit manipulation. *J Neurosci* 30: 9117-9126, 2010.

**Gomi H, and Osu R**. Task-dependent viscoelasticity of human multijoint arm and its spatial characteristics for interaction with environments. *J Neurosci* 18: 8965-8978, 1998.

**Salimi I, Hollender I, Frazier W, and Gordon AM**. Specificity of internal representations underlying grasping. *J Neurophysiol* 84: 2390-2397, 2000.

**Shadmehr R, Brandt J, and Corkin S**. Time-dependent motor memory processes in amnesic subjects. *J Neurophysiol* 80: 1590-1597, 1998.

**Smith MA, Ghazizadeh A, and Shadmehr R**. Interacting adaptive processes with different timescales underlie short-term motor learning. *PLoS Biol* 4: e179, 2006.

**Witney AG, Goodbody SJ, and Wolpert DM**. Learning and decay of prediction in object manipulation. *J Neurophysiol* 84: 334-343., 2000.

**Witney AG, and Wolpert DM**. Spatial representation of predictive motor learning. *J Neurophysiol* 89: 1837-1843, 2003.

**Zhang W, Gordon AM, Fu Q, and Santello M**. Manipulation after object rotation reveals independent sensorimotor memory representations of digit positions and forces. *J Neurophysiol* 103: 2953-2964, 2010.
